# *In vivo* validation of the palmitoylation cycle as a therapeutic target in *NRAS*-mutant cancer

**DOI:** 10.1101/2025.03.20.644389

**Published:** 2025-03-21

**Authors:** Matthew Decker, Benjamin J. Huang, Timothy Ware, Christopher Boone, Michelle Tang, Julia Ybarra, Aishwarya C. Ballapuram, Katrine A. Taran, Pan-Yu Chen, Marcos Amendáriz, Camille J. Leung, Max Harris, Karensa Tjoa, Henry Hongo, Sydney Abelson, Jose Rivera, Nhi Ngo, Dylan M. Herbst, Radu M. Suciu, Carlos Guijas, Kimia Sedighi, Taylor Andalis, Elysia Roche, Boer Xie, Yunlong Liu, Catherine C. Smith, Elliot Stieglitz, Micah J. Niphakis, Benjamin F. Cravatt, Kevin Shannon

**Affiliations:** 1 Department of Pediatrics, University of California, San Francisco, San Francisco, CA, USA.; 2 Helen Diller Family Comprehensive Cancer Center, University of California, San Francisco, San Francisco, CA, USA.; 3 Department of Chemistry, The Scripps Research Institute, La Jolla, CA, USA.; 4 Lundbeck La Jolla Research Center, Inc., San Diego, CA, USA.; 5 Center for Center for Medical Genomics, Department of Medical and Molecular Genetics, Indiana University School of Medicine, Indianapolis, IN, USA.; 6 Department of Medicine, University of California, San Francisco, San Francisco, CA, USA.

## Abstract

Normal and oncogenic Ras proteins are functionally dependent on one or more lipid modifications^1,2^. Whereas K-Ras4b farnesylation is sufficient for stable association with the plasma membrane, farnesylated H-Ras, K-Ras4a, and N-Ras traffic to the Golgi where they must undergo palmitoylation before regulated translocation to cell membranes. N-Ras palmitoylation by the DHHC family of palmitoyl acyl transferases (PATs) and depalmitoylation by ABHD17 serine hydrolases is a dynamic process that is essential for the growth of acute myeloid leukemias (AMLs) harboring oncogenic *NRAS* mutations^3–6^. Here, we have tested whether co-targeting ABHD17 enzymes and Ras signal output would cooperatively inhibit the proliferation and survival of *NRAS*-mutant AMLs while sparing normal tissues that retain K-Ras4b function. We show that ABD778, a potent and selective ABHD17 inhibitor with *in vivo* activity, selectively reduces the growth of *NRAS*-mutant AML cells *in vitro* and is synergistic with the allosteric MEK inhibitor PD0325901 (PD901)^7,8^. Similarly, ABD778 and PD901 significantly extended the survival of recipient mice transplanted with three independent primary mouse AMLs harboring an oncogenic *Nras*^*G12D*^ driver mutation. Resistant leukemias that emerged during continuous drug treatment acquired by-pass mutations that confer adaptive drug resistance and increase mitogen activated protein kinase (MAPK) signal output. ABD778 augmented the anti-leukemia activity of the pan-PI3 kinase inhibitor pictilisib^9^, the K/N-Ras^G12C^ inhibitor sotorasib^10^, and the FLT3 inhibitor gilteritinib^11^. Co-treatment with ABD778 and gilteritinib restored drug sensitivity in a patient-derived xenograft model of adaptive resistance to FLT3 inhibition. These data validate the palmitoylation cycle as a promising therapeutic target in AML and support exploring it in other *NRAS*-mutant cancers.

Somatic *NRAS* mutations occur in 3–5% of adult and pediatric cancers and are most prevalent in hematologic malignancies, melanoma, and thyroid cancer^12,13^. In addition, *NRAS* mutations are key drivers of *de novo* and adaptive resistance to BCL-2, IDH1/2, and FLT3 inhibitors in acute myeloid leukemia (AML) and in melanomas that relapse after Raf kinase inhibitor treatment^14–17^. There are no approved targeted therapies for *NRAS*-mutant cancers.

Sequential farnesylation and palmitoylation of the N-Ras C-terminal hypervariable region (HVR) mediates stable association with cell membranes and is required for efficient N-Ras activation and downstream signaling^1^. Whereas farnesylation is irreversible, the N-Ras palmitoylation/depalmitoylation cycle is highly dynamic^18^. The depalmitoylation reaction maintains a kinetic trap in the Golgi apparatus, where addition of the palmitoyl group significantly increases lipophilic affinity of N-Ras and locks it in place^19^. Once it is trapped in the Golgi via palmitoylation, N-Ras undergoes anterograde trafficking to the plasma membrane in a complex with VPS35^20^. Inhibiting either the “on” or the “off” reaction results in N-Ras mislocalization and impaired downstream signaling^21^.

The essential role of farnesylation in Ras transformation stimulated drug discovery programs in the 1990s that generated potent and selective farnesyl transferase inhibitors^22^. Unfortunately, these compounds proved ineffective in most *RAS*- mutant cancers due to by-pass geranylgeranylation of the C-terminal cysteines of N-Ras and K-Ras^22–25^. After this disappointing experience, the pharmaceutical industry largely abandoned efforts to develop new approaches for inhibiting the lipid modification of N-Ras and K-Ras.

The palmitoylation cycle has theoretical advantages as a therapeutic target over farnsylation/geranylgeranylation in *NRAS*-mutant cancers. No compensatory lipid modification for N-Ras palmitoylation has yet been identified. In addition, while the palmitoylation cycle is required for proper localization and activity of N-Ras, K-Ras4a, and H-Ras, the K-Ras4b HVR has a lipophilic poly-lysine domain that enhances membrane affinity, which obviates the requirement for a second lipid modification. In principle, this “biologic therapeutic index” could allow healthy tissues to tolerate chemical inhibition of the N-Ras palmitoylation cycle by signaling through K-Ras4b. Finally, we and others showed that introducing a cysteine-to-serine mutation (C181S) into the N-Ras HVR abrogates palmitoylation, inhibits the growth of *NRAS*-mutant AML cell lines in a genotype-specific manner, and suppresses myeloid transformation by oncogenic *NRAS in vitro* and *in vivo*^3,5,6,26^.

The ABHD17 family of serine hydrolases has been identified as the principal N-Ras depalmitoylases^4,5^. We previously reported that a pan-ABHD17 inhibitor ABD957 selectively reduces growth and suppresses MAPK signaling in *NRAS*-mutant AML cell lines^5^. ABD957 also showed synergistic activity with the allosteric MEK inhibitor PD0325901 (PD901) in these models. Based on these promising *in vitro* data, we pursued the development of an ABHD17 inhibitor suitable for *in vivo* studies, culminating in the discovery of a potent, selective and *in vivo*-active ABHD17 inhibitor, ABD778. Here we show that co-treatment with ABD778 and PD901 selectively inhibits the growth of AML cell lines and primary juvenile myelomonocytic leukemias harboring oncogenic *NRAS* mutations, markedly extends survival in recipient mice transplanted with *Nras*-mutant AMLs and drives the emergence of adaptive resistance *in vivo*. Additionally, ABD778 exhibited synergy with the class 1 PI3 kinase inhibitor pictilisib^9^, the K/N-Ras^G12C^ inhibitor sotorasib^10^, and the FLT3 inhibitor gilteritinib in mouse and human AML cells expressing oncogenic N-Ras proteins. Finally, co-treatment with ABD778 and gilteritinib suppressed the growth of a patient-derived xenograft (PDX) model of adaptive resistance to FLT3 inhibition.

## RESULTS

### Development of a pan-ABHD17 inhibitor with *in vivo* activity

**Figure 1.**
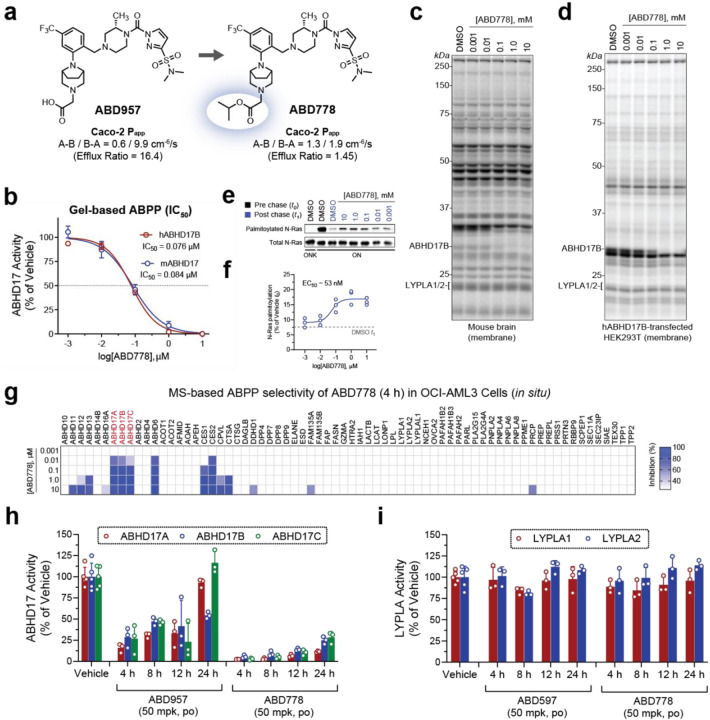
Discovery and characterization of ABD778 as an ABHD17 inhibitor with *in vivo* activity. **a,** Structures of ABD957 and ABD778 and corresponding Caco-2 permeability data highlighting improved passive permeability and reduced efflux of ABD778. **b**, Half maximal inhibitor concentration (IC_50_) curves for ABD778 against endogenous mouse ABHD17 and recombinantly expressed human ABHD17B was measured by gel-based activity based proteomic profiling (ABPP). Data presented as mean values ± s.e.m (n = 2–3). **c,d,** Competitive gel-based ABPP profiling of ABD778 (0.001–10 μM) in mouse brain membrane (panel c) and in HEK293T cells transduced with a cDNA encoding human ABHD17B (panel d) showing selective inhibition of ABHD17 over FP-Rh-labeled mouse and human serine hydrolases. **e**, ABD778 partially inhibits N-Ras depalmitoylation. Representative gel measuring N-Ras^G12D^ palmitoylation by 17-ODYA in the presence of varying concentrations of ABD778 (upper panel). N-Ras^G12D^ was immunoprecipitated via GFP and the degree of palmitoylation visualized by rhodamine azide attached via copper(I)-catalyzed click chemistry to the alkyne of 17-ODYA. Total N-Ras content was measured by western blotting of GFP enrichments (bottom panel). As expected, 17-ODYA labeling was not observed in OCI-AML3/ONK cells in which the N-Ras hypervariable domain (HVR) was replaced by the K-Ras4b HVR. **f**, Calculated IC_50_ values for ABD778 stabilization of N-Ras^G12D^ palmitoylation as measured in **e** (n = 3 per group). **g,** *In situ* MS-based ABPP data from OCI-AML3 cells that were exposed to ABD778 (0.001–10 μM) for 4 h demonstrating ABHD17A/B/C inhibition at low nanomolar concentrations and selectivity across depalmitoylases LYPLA1, LYPLA2 and ABHD10. Data plotted represent mean competition from three biological replicates. **h**,**i**, *In vivo* target engagement for ABHD17A/B/C (panel h) and LYPLA1/2 (panel i) following ABD957 or ABD778 administration. C57Bl/6 mice were dosed with vehicle, ABD957 (50 mg/kg, po) or ABD778 (50 mg/kg, po). Spleen tissue was collected 4, 8, 12 and 24 h after compound administration and analyzed by targeted MS-ABPP using FP-biotin enrichment of serine hydrolase enzymes and parallel reaction monitoring (PRM) to detect and quantify unique diagnostic peptides from each ABHD17 and LYPLA enzyme. ABD778 provided near complete and sustained blockade of ABHD17 enzymes over 24 h whereas ABD957 showed transient inhibitory activity. Both compounds maintained selectivity over LYPLA1 and LYPLA2. Data plotted represent the median from biological replicates, and error bars represent s.d. (n = 3–5).

Previously, we reported the development of a pan-ABHD17 covalent inhibitor ABD957 (Fig. 1a), which potently inhibited ABHD17 enzymes in cells and featured high selectivity over depalmitoylases including LYPLA1/2, PPT1 and ABHD10^5^. While ABD957 provided a useful tool to study ABHD17 enzymes in cellular contexts, this compound was found to have low permeability (Caco-2 P_app_ (A-B) = 0.6 cm^−6^/s) and high efflux (efflux ratio = 16.4) in Caco-2 cells which we suspected would limit its exposure *in vivo* (Fig. 1a). We reasoned that reducing the polarity of ABD957 by esterifying its carboxylic acid would improve these properties and potentially provide a prodrug of ABD957 in the event of *in vivo* ester hydrolysis. The isopropyl ester analog of ABD957, here termed ABD778, exhibited enhanced passive permeability (Caco-2 P_app_ (A-B) = 1.3 cm^−6^/s) and minimal efflux (efflux ratio = 1.45) (Fig. 1a). We next determined if this modification altered the potency and selectivity of ABD778 in mouse brain proteomes and hABHD17B-transfected HEK293T cell lysates using gel-based activity-based protein profiling (ABPP) where over 25 native serine hydrolases can be evaluated owing to their reactivity with the active site-directed fluorophosphonate rhodamine (FP-Rh) probe^27,28^. This analysis revealed that ABD778 maintained high potency against both human and mouse ABHD17 enzymes (IC_50_ = 0.076 and 0.084 μM, respectively) (Fig. 1b) and demonstrated high specificity for ABHD17 enzymes across the detectable serine hydrolases in both mouse (Fig. 1c) and human (Fig. 1d) proteomes. Deeper selectivity profiling was then performed across >90 mouse and human serine hydrolases in mouse brain and OCI-AML3 (a human *NRAS*-mutant AML cell line) proteomes using mass spectrometry (MS)-ABPP^29^. A few additional off-targets were detected, including ABHD6 and several carboxyesterases (Extended Data Fig. 1a, 1b). Notably, these primary off-targets are common to many covalent serine hydrolase inhibitors^30^ and were accounted for in previous experiments with ABD957 through the use of inactive control compounds^5^, which revealed that these off-targets do not substantially contribute to N-Ras depalmitoylation.

We next evaluated the activity of ABD778 on N-Ras palmitoylation in AML cells. OCI-AML3/ON and OCI-AML3/ONK cells are isogenic OCI-AML3 sublines that were engineered to be dependent on a green fluorescent protein(GFP)-labeled mouse N-Ras^G12D^ (ON cells) or a fusion protein in which the N-Ras HVR was replaced by the K-Ras4b HVR (ONK cells)^5^. Pulse-chase labeling with the clickable palmitate analog 17-octadecynoic acid (17-ODYA) revealed selective inhibition of N-Ras^G12D^ depalmitoylation by ABD778 in OCI-AML3/ON cells at a similar EC_50_ value as previously observed for ABD957 (Figs. 1e, 1f)^5^. We additionally confirmed that ABD778 potently inhibited all ABHD17 isoforms in OCI-AML3 cells while maintaining a similar selectivity profile as observed in cell lysates (Fig. 1g).

To evaluate the suitability of ABD778 for *in vivo* studies, we dosed C57Bl/6 mice with vehicle, ABD957 (50 mg/kg, og), or ABD778 (50 mg/kg, og) and measured ABHD17 target engagement 4, 8, 12 and 24 hours (h) later in spleen tissue using targeted MS-ABPP. ABD778 produced maximal inhibition of all ABHD17 isoforms at 4 h and maintained >50% occupancy over the 24 h time-course (Fig. 1h). In contrast, ABD957 only partially inhibited ABHD17 enzymes at early time points (4–8 h) and this effect was not sustained at later time points (24 h; Fig. 1h). Consistent with their ABPP selectivity profiles *in vitro*, neither ABD957 or ABD778 inhibited LYPLA1 and LYPLA2 *in vivo* (Fig. 1i). While ABD778 could serve as a prodrug that is converted through esterolysis to ABD957, we observed limited evidence for this conversion *in vivo* (Extended Data Fig. 1c). This finding, together with ABD778’s potent inhibition of ABHD17 enzymes *in vitro*, suggests that ABD778 itself, rather than ABD957 derived from ABD778 hydrolysis, is likely the active drug in mice. Together, these data support the use of ABD778 as a selective and *in vivo*-active pan-ABHD17 inhibitor.

### ABD778 selectively reduces the growth of *NRAS*-mutant leukemia cells and is synergistic with PD901 *in vitro*

**Figure 2.**
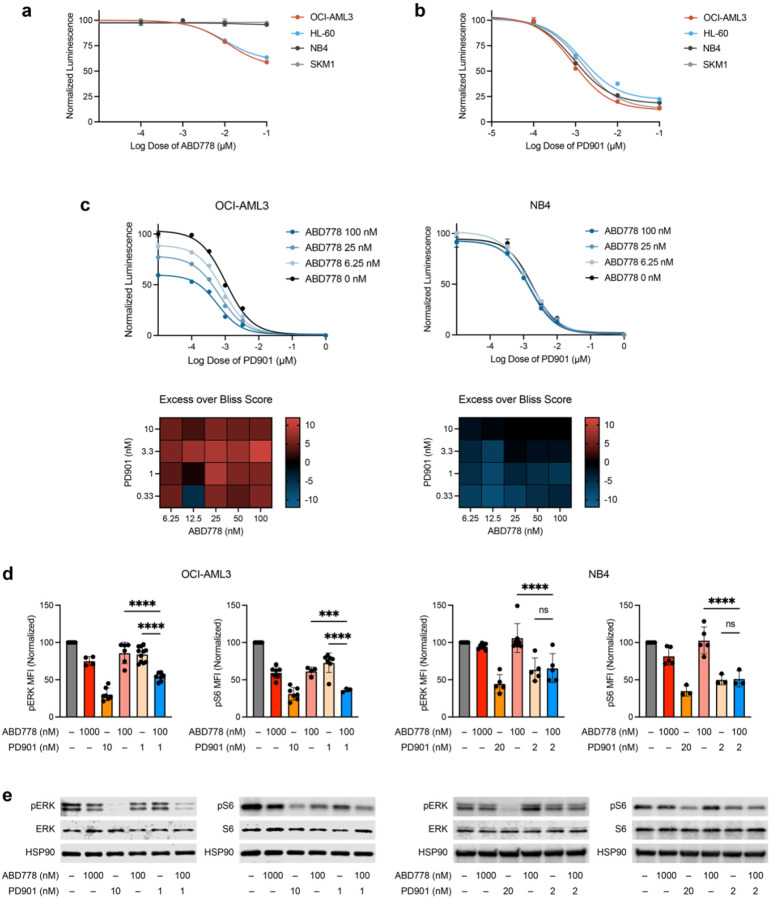
Selective activity of ABD778 and synergy with PD901 in *NRAS*-mutant AML cell lines. **a, b,** The proliferation of *NRAS* mutant OCI-AML3 and HL-60 cells and of *KRAS* mutant NB4 and SKM1 cells were assessed 72 h after exposure to ABD778 (panel a) or PD901 (panel b) using Cell Titer-Glo. **c**, Bliss independence analysis demonstrating synergistic growth inhibition over a broad range of ABD778 and PD901 concentrations in OCI-AML3 (left), but not NB4 (right), cells. Heatmaps display calculated synergy scores from strongly positive (red) to negative (blue). The data presented in panels a–c data generated in triplicate and were replicated in at least two additional independent experiments. **d,** Phosphorylated ERK (pERK) and S6 (pS6) levels were measured using phospho-flow cytometry in OCI-AML3 (left) and NB4 (right) cells exposed to ABD778 and/or PD901 for 4h at the doses shown. Mean fluorescence index (MFI) values were normalized to 100% of the DMSO control and pooled for statistical analysis. n = 4–11; *** - p < 0.001; **** - p < 0.0001. **e,** pERK and pS6 levels were measured by Western blotting in OCI-AML3 (left) and NB4 (right) cells that were exposed to DMSO, ABD778, PD901, or both drugs as in panel d. The data presented were generated in (panel d) or are representative of (panel e) at least three independent experiments.

In Cell Titer-Glo (CTG) assays, ABD778 reduced the growth of OCI-AML3 and another human AML cell line (HL-60) harboring oncogenic *NRAS* mutations, but not of the *KRAS*-mutant AML cell lines NB4 and SKM1 (Figs. 2a, Extended Data Fig. 2). By contrast, PD901 non-selectively suppressed the proliferation of both *NRAS*- and *KRAS*-mutant AML cells (Figs. 2b, Extended Data Fig. 2). Exposing OCI-AML3/ON and ONK cells to ABD778 confirmed that growth inhibition is dependent on the N-Ras HVR and correlated with reduced ERK phosphorylation (Extended Data Fig. 3). We hypothesized that co-treatment with PD901 might augment the genotype-specific activity of ABD778. Indeed, ABHD17 and MEK inhibition synergistically inhibited the growth of *NRAS*-mutant, but not *KRAS*-mutant, AML cells as assessed by the Bliss independence method (Figs. 2c, Extended Data Fig. 2). Consistent with these data, phosphor-flow cytometric and Western blot analyses showed that ABD778/PD901 co-treatment cooperatively reduced constitutively elevated levels of phosphorylated ERK (pERK) in OCI-AML3 and HL-60 cells, but not in NB4 or SKM1 cells (Figs. 2d, 2e, Extended Data Fig. 4). Phosphorylated S6 (pS6) levels were cooperatively decreased in OCI-AML3 cells after exposure to ABD778 and PD901, but not in HL-60, NB4, or SKM1 cells (Figs. 2d, 2e, Extended Data Fig. 4). The observed pattern of pERK and pS6 repression is consistent with enhanced MAPK pathway inhibition as the principal mechanism of ABD778/PD901 synergy.

**Figure 3.**
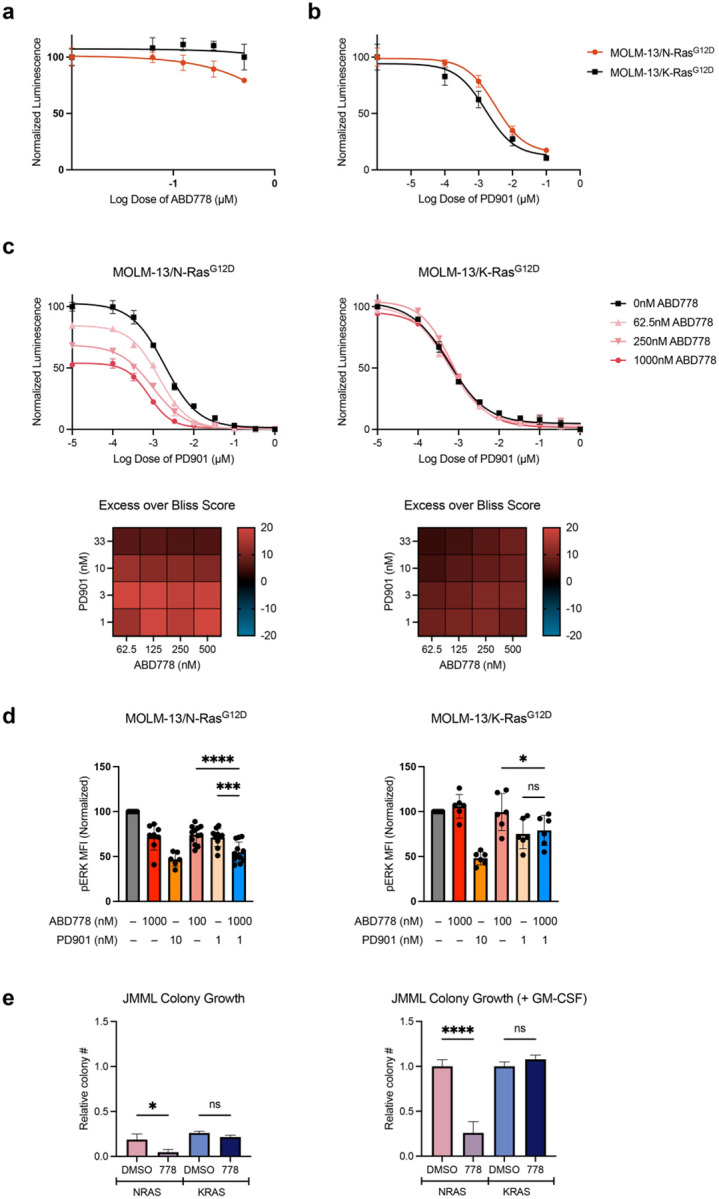
Activity of ABD778 in isogenic MOLM-13 cells expressing oncogenic N-Ras^G12D^ or K-Ras^G12D^ and JMML patient samples. **a,b,** MOLM-13 cells that were treated with doxycycline (Dox) for 24h to induce exogenous N-Ras^G12D^ or K-Ras^G12D^ expression as described previously^31,32^. were exposed a range of ABD778 and PD901 doses for 72h. Proliferation was assessed by Cell Titer-Glo. **c,** Bliss independence analysis of MOLM-13/N-Ras^G12D^ (left) and MOLM13/K-Ras^G12D^ (right) cells that were treated with doxycycline (Dox) for 24h and then exposed a range of ABD778 and PD901 doses for 72h as described in Figure 2. Heatmaps display calculated synergy scores from strongly positive (red) to negative (blue). **d,** pERK levels were measured by phospho-flow cytometry in MOLM13/N-Ras^G12D^ (left) and MOLM13/K-Ras^G12D^ (right) cells that were exposed to the ABD778 and/or PD901 doses shown for 4h. Mean fluorescence index (MFI) values were normalized to 100% in cells that were treated with the DMSO vehicle and were pooled for statistical analysis. The data shown in Figures 3a–3d are from at least three independent experiments. n = 6–12; *- p < 0.05; ***- p < 0.001; **** - p < 0.0001. **e**, CFU-GM colonies were grown in methylcellulose medium from JMML patient samples harboring *NRAS* or *KRAS* mutations (n = 3 of each genotype) with and without ABD778 (1 μM). Cytokine independent CFU-GM colony growth is shown on the left and the number of CFU-GM colonies observed in cultures containing a saturating dose of GM-CSF (10 ng/mL) is shown on the right. Colony growth was normalized to 100% (1.0) for *NRAS*-mutant and *KRAS*-mutant patient samples grown in GM-CSF, respectively. Note that the GM-CSF markedly augmented CFU-GM growth in JMML cells of both genotypes and that ABD778 robustly suppressed the growth of *NRAS* mutant leukemias.

We next deployed a “switchable” isogenic model in which exposing *FLT3*-*ITD* mutant MOLM-13 AML cells to the FLT3 inhibitor quizartinib abrogates oncogenic signaling and renders these cells dependent on exogenous doxycycline-regulated mutant Ras protein expression for proliferation and survival^31,32^. In this system, ABD778 reduced the growth of cells engineered to express N-Ras^G12D^ (MOLM-13/N-Ras^G12D^), but not K-Ras^G12D^ (MOLM-13/K-Ras^G12D^) (Fig. 3a). By contrast, MOLM-13/N-Ras^G12D^ and MOLM-13/K-Ras^G12D^ cells exhibited similar sensitivities to PD901 (calculated 50% inhibitory concentration (IC_50_) values 3 nM and 1.6 nM, respectively) (Fig 3b). Bliss independence analysis revealed genotype-selective growth inhibition in MOLM-13/N-Ras^G12D^ cells over a range of ABD778 and PD901 concentrations that correlated with reduced pERK levels in response to ABD778/PD901 (Figs. 3c, 3d).

JMML is an aggressive myeloproliferative neoplasm initiated by mutations in *NRAS, KRAS*, and other genes that constitutively increase Ras signal output^33,34^. Cytokine-independent colony-forming unit granulocyte-macrophage (CFU-GM) progenitor growth and hypersensitivity to low concentrations of granulocyte-macrophage colony stimulating factor (GM-CSF) in methylcellulose cultures are cellular hallmarks of JMML that are also observed in bone marrow cells from *Kras*^*G12D*^ and *Nras*^*G12D*^ knock-in mice^35–37^. As expected, primary *KRAS*- and *NRAS*-mutant CD34^+^ JMML cells exhibited cytokine-independent CFU-GM colony growth, which was augmented by a saturating dose of GM-CSF (Fig. 3e; Extended Data Table 1). ABD778 significantly reduced both cytokine-independent and GM-CSF-stimulated colony growth of JMML cells with *NRAS* mutations but had no effect on *KRAS*-mutant cells (Fig. 3e).

### *In vivo* efficacy of MEK and ABHD17 inhibition in primary *Nras*^*G12D*^ AMLs

**Figure 4.**
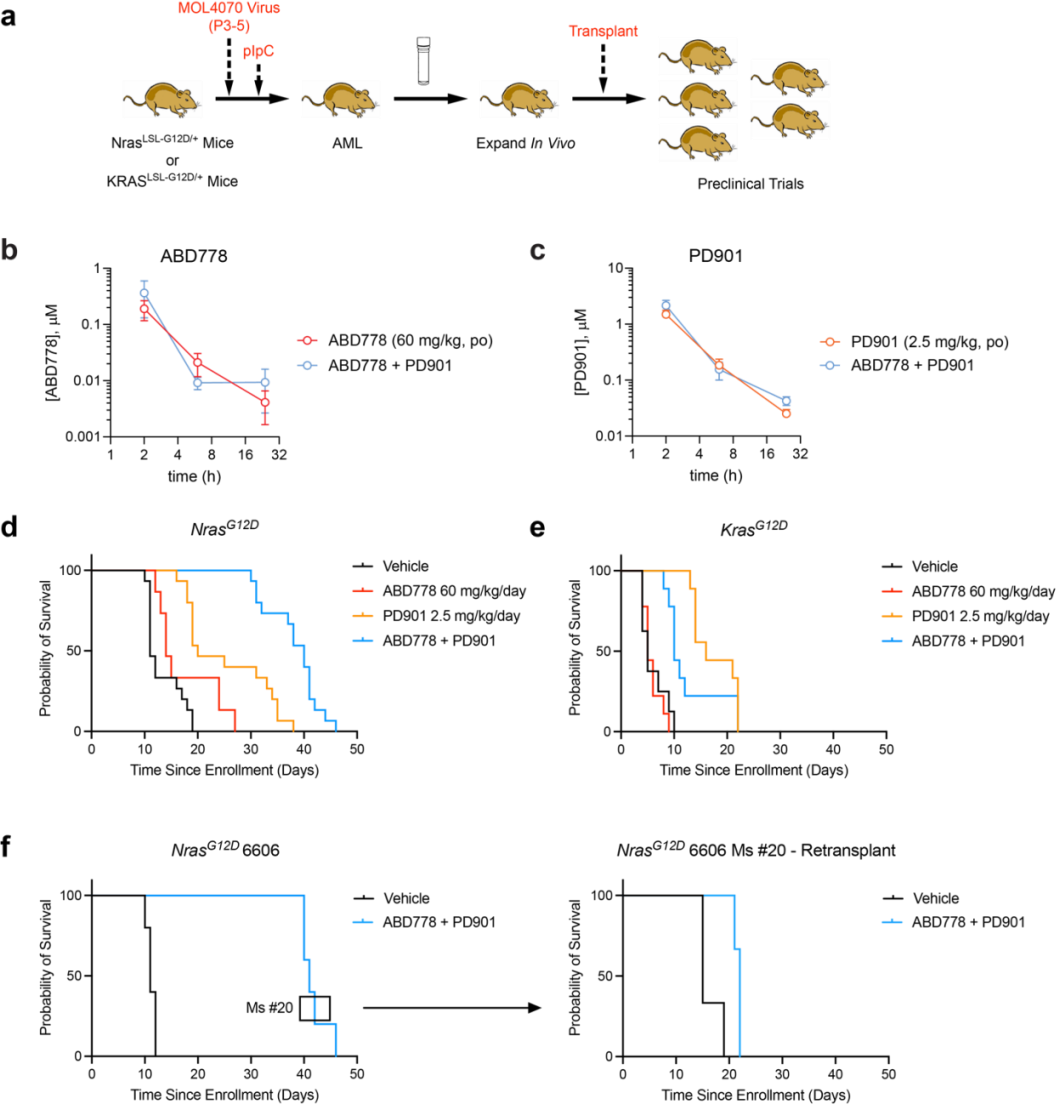
Efficacy of *in vivo* ABD778 and PD901 treatment in primary *Nras*^*G12D*^ and *Kras*^*G12D*^ AMLs. **a,** Transplantable primary AMLs were generated by injecting neonatal wild-type (WT), *Mx1-Cre; Nras*^*LSL-G12D/+*,^ or *Mx1-Cre; Kras*^*LSL-G12D/+*,^ mutant mice with the MOL4070LTR retrovirus followed by a single dose of polyI-polyC at weaning to induce *Nras*^*G12D*^ or *Kras*^*G12D*^ expression from the endogenous loci as described previously^37,41^. Preclinical trials are performed by expanding cryopreserved leukemia cells *in vivo* and transplanting 1–2 ×10^6^ bone marrow cells into 10–20 congenic recipients^38–40^. **b**, c Blood concentrations of ABD778 (panel b) and PD901 (panel c) following a single dose of ABD778 (60 mg/kg, po), PD901 (2.5 mg/kg, po) or both compounds dosed 1 h apart in mice. Blood was collected 2, 6, and 24 h after compound administration and analyzed LC-MS/MS. Coadministration of ABD778 and PD901 (blue line) provides similar compound exposures compared with single-agent dosing (red and yellow lines for ABD778 and PD901, respectively). **d**, Cumulative probability of survival of mice transplanted with *Nras*^*G12D*^ AMLs 6606, 6695, and 6768 that were treated with the control vehicle (black line), ABD778 (red line), PD901 (yellow line), or PD901 + ABD778 (blue line). GraphPad Prism software was used to generate Kaplan-Meier survival curves (primary endpoint) and statistical significance between individual trial arms was calculated using a two-tailed Log-Rank test. These values are presented in the text. **e**, Cumulative probability of survival of mice transplanted with *Kras*^*G12D*^ AMLs 21B and 63A that received the control vehicle (black line), ABD778 (red line), PD901 (yellow line), or PD901 + ABD778 (blue line). Statistical analysis was performed as described in panel b and significant differences reported in the text. **f**, The left panel shows the survival of 5 recipient mice of *Nras*^*G12D*^ AML 6606 that were treated with ABD778 + PD901 in the initial trial. Leukemia cells collected at euthanasia from the recipient shown in the black box were transplanted into secondary recipients and re-treated with either control vehicle or ABD778 + PD901 (n = 3 mice per group). The right panel shows the survival of these mice.

Transplantable mouse AMLs generated by retroviral insertional mutagenesis in *Kras*^*G12D*^ and *Nras*^*G12D*^ mice are robust models for performing controlled preclinical trials for assessing drug efficacy and identifying mechanisms of adaptive resistance^38–40^. In this system, retroviral integrations in proto-oncogenes such as *Ev11, Myb*, and *Sox4* cooperate with subsequent *Nras*^*G12D*^or *Kras*^*G12D*^ expression^37,40,41^ in leukemogenesis. This reflects the common pathogenic sequence in human AML whereby *RAS* mutations cooperate with antecedent genetic drivers^42,43^. As in human patients, adaptive resistance can result from the outgrowth of pre-existing clones or the acquisition of somatic mutations during treatment^39,40,44^. Cryopreserved primary leukemia cells are first expanded in one or more “factory” mice and then transplanted into cohorts of immunocompetent congenic recipients that are treated with vehicle or drug(s) (Fig. 4a). In previous studies, PD901 had modest single-agent activity in *Kras*^*G12D*^ and *Nras*^*G12D*^ leukemias with only one of 10 independent AMLs acquiring adaptive resistance during continuous drug treatment (Extended Data Table 2)^38,39^.

Before comparing single-agent ABD778 and PD901 and combination treatment in mice, we asked if PD901 alters ABD778 blood levels or ABHD17 target engagement in peripheral tissues. Implementing a more effective protocol for suspending PD901 allowed us to use a lower dose than in previous preclinical trials (2.5 versus 5 mg/kg day)^38–40^. Spleen, kidney and blood samples were collected 2, 6 and 24 h after administration of vehicle, ABD778 (60 mg/kg, og), PD901 (2.5 mg/kg, og) or both compounds dosed 1 h apart. ABD778 and PD901 levels in the blood were comparable at each time point measured whether dosed as single agents or in combination (Figs. 4b, 4c). Accordingly, ABD778 dosed alone or in combination with PD901 produced robust and similar target engagement across ABHD17 isoforms in spleen and kidney (Extended Data Fig. 5).

Having confirmed minimal *in vivo* drug-drug interactions between ABD778 and PD901, we sequentially transplanted five groups of 20 mice each with *Nras*-mutant AMLs 6606, 6695, and 6768 or with *Kras*-mutant leukemias 21B and 63A^37–39^. These recipients were treated continuously with control vehicle, ABD778 (60 mg/kg/day), PD901 (2.5 mg/kg/day), or this combination (n = 5 mice per group) by oral gavage. Kaplan-Meier analysis showed that ABD778 modestly improved the survival of mice transplanted with *Nras*-mutant, but not *Kras*-mutant, AMLs (p = 0.0168) (Figs. 4d, 4e). Consistent with previous preclinical studies^38,39^, PD901 treatment extended survival in recipients of *Nras*^*G12D*^ and *Kras*^*G12D*^ AMLs by approximately two-fold (p < 0.0001 for *Nras*^*G12D*^ mice (n = 15); and p < 0.0001 for *Kras*^*G12D*^ mice (n=10), respectively) (Figs. 4d, 4e, Extended Data Tables 2, 3). Co-treatment with ABD778 and PD901 further prolonged survival in mice engrafted with *Nras*^*G12D*^ leukemias from ~20 to ~40 days (p < 0.0001 versus vehicle; p < 0.0001 versus PD901 alone) (Fig 4d). By contrast, there was no significant difference in the survival of recipients of *Kras*^*G12D*^ leukemias that were assigned to the PD901 and PD901/ABD778 treatment arms (Fig. 4e). A similar pattern of drug responses seen in the pooled analysis of 100 recipient mice was also evident in each independent AML (Extended Data Fig. 6).

Single-agent and combination treatment with ABD778 was well tolerated with minimal weight loss that was similar to the vehicle and PD901 groups (Extended Data Fig. 7). All recipient mice died with progressive AML characterized by blood leukocytosis, anemia, and splenomegaly (Extended Data Fig. 7). We re-transplanted *Nras*^*G12D*^ AMLs isolated from mice that initially responded to combination treatment and retreated them with ABD778 and PD901 *in vivo*. Secondary recipients had greatly reduced survival in comparison to mice enrolled in the initial trial, verifying intrinsic drug resistance in leukemias that emerged after an initial response to the ABD778/PD901 combination (Fig. 4f, Extended Data Fig. 8).

### Emergent on-mechanism mutations cause resistance to ABD778/PD901

To identify candidate resistance mechanisms, we performed whole exome sequencing (WES) of *Nras*^*G12D*^ AMLs isolated at euthanasia from recipient mice treated with either control vehicle or ABD778/PD901. As in relapsed human AMLs^44^, vehicle-treated and resistant leukemias shared multiple “founder” mutations (Extended Data Table 4). Interestingly, leukemia cells isolated from multiple recipients of AML 6606 showed a *Nras*^*G12D*^ variant allele frequency (VAF) of ~80–90% after ABD778/PD901 treatment versus ~60% in mice that received the control vehicle (Extended Data Table 4). This likely reflects outgrowth of a pre-existing minor clone with uniparental disomy for the mutant *Nras* allele^37,45^. WES also uncovered mutations identified in human cancers at diagnosis or after treatment with signal transduction inhibitors at variant allele frequencies (VAFs) of 3–17% that included *Flt3*^*D835E*^*, Kras*^*A146T*^*, Braf*^*G466E*^, and *Pik3ca*^*F977Y* 46–49^ (Extended Data Table 4). The observation that each of these mutations were detected in one of five recipient mice assigned to receive ABD778/PD901 suggests that they were acquired during treatment.

**Figure 5.**
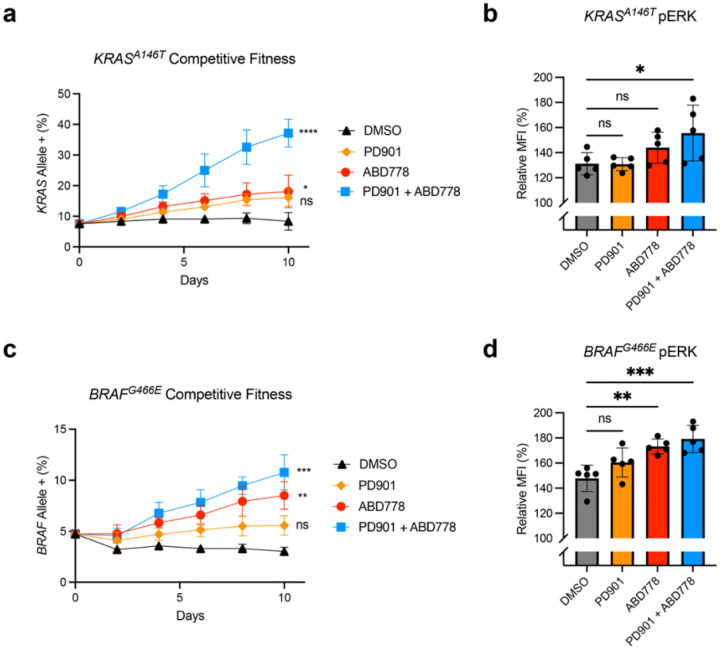
Mutations detected at euthanasia in recipient of *Nras*^*G12D*^ AMLs confer resistance to ABD778 and PD901. **a,** Percentages of mCherry-positive (mCherry^+^) OCI-AML3 cells that were transduced with a *mCherry-KRAS*^*A146T*^ expression vector and tracked over 10 days of exposure to either DMSO (black), PD901 (orange), ABD778 (red), or PD901 and ABD778 (blue). Cells were analyzed and replated with fresh drug added every 48h. These data were generated from three independent biological replicates. Day 10 time points were pooled for statistical analysis. **** p < 0.0001; * p < 0.05. **b.** Relative pERK levels of *mCherry-KRAS*^*A146T*^ -transduced OCI-AML3 cells compared to non-transduced cells within the same drug treatment well. Cells in treatment wells were exposed to DMSO, PD901, ABD778, or both drugs for 4h. n = 5; * p < 0.05. **c.** Percentages of mCherry^+^ OCI-AML3 cells that were transduced with a *mCherry*-*BRAF*^*G466E*^ and monitored for 10 days as in panel a. Data from three independent biological experiments. Day 10 time points were pooled for statistical analysis. *** p < 0.001; ** p < 0.01. **d,** Relative pERK levels of *mCherry*-*BRAF*^*G466E*^-transduced OCI-AML3 cells compared to non-transduced cells within the same drug treatment well as in panel c. n = 5; *** p < 0.001; ** p < 0.01.

To model the outgrowth of a minor drug-resistant clone, we infected OCI-AML3 cells with lentiviral vectors encoding the respective mutant proteins fused to a mCherry reporter gene at a low multiplicity of infection. Transduced cells were then exposed to vehicle, PD901, ABD778, or the PD901/ABD778 combination. *Kras*^*A146T*^ expression resulted in a significant competitive growth advantage upon drug, but not vehicle, exposure that was most pronounced in cells treated with ABD778 and PD901, with the *Kras*^*A146T*^ clone expanding to nearly 40% of the total population after 10 days in culture. Outgrowth was characterized by elevated pERK levels in cells expressing K-Ras^A146T^ (Figs. 5a, 5b). Similarly, *Braf*^*G466E*^ expression increased the competitive fitness of transduced OCI-AML3 cells exposed to ABD778 or ABD778/PD901, which was associated with MAPK pathway activation (Figs. 5c, 5d). We conclude that primary *Nras*^*G12D*^ AMLs that relapse after combination treatment with ABD778 and PD901 treatment acquire mutations that cause adaptive drug resistance and restore oncogenic signaling.

### ABD778 augments the anti-leukemia activity of other Ras pathway inhibitors and overcomes adaptive resistance to FLT3 inhibition

We reasoned that the synergistic activity of ABD778 and PD901 in *NRAS/Nras*-mutant human and mouse leukemias might extend to additional targeted inhibitors of Ras signaling. In a previous preclinical study, the class 1 PI3 kinase inhibitor pictilisib (also known as GDC-0941) failed to significantly extend the survival of recipient mice transplanted with *Nras*^*G12*D^ AMLs 6606, 6695, and 6768^38^. Interestingly, however, Bliss independence analysis showed synergistic growth inhibition in OCI-AML3 cells exposed to ABD778 and pictilisib (Extended Data Fig. 9). We did not observe synergistic growth inhibition in NB4 cells. Western blot analysis revealed equivalent reduction of Akt phosphorylation in OCI-AML3 and NB4 cells after short-term exposure to pictilisib or ABD778/pictilisib. However, pERK and pS6 levels were cooperatively and selectively decreased in OCI-AML3 cells treated with ABD778/pictilisib, which is consistent with ABD778 inhibition of compensatory MAPK activity in pictilisib-treated cells^50^ (Extended Data Fig. 10).

**Figure 6.**
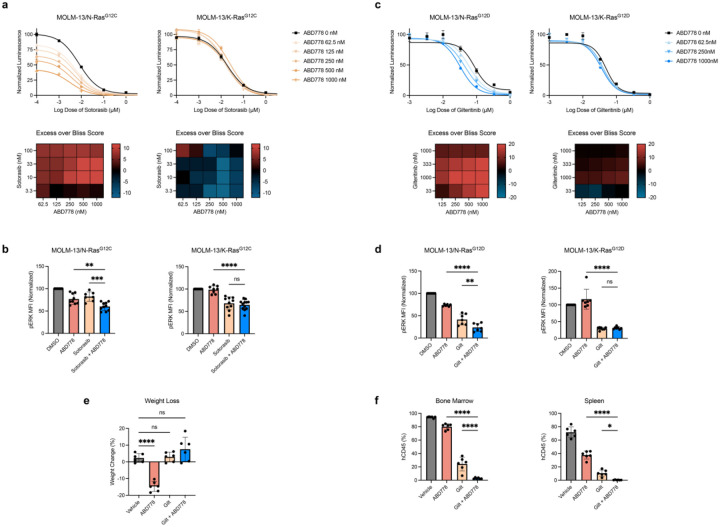
ABD778 cooperates with chemical inhibitors of PI3 kinase, Ras^G12C^, and FLT3 to suppress the growth of *NRAS*-mutant AML cells. **a,** Bliss independence analysis of MOLM-13/N-Ras^G12C^ (left) and MOLM13/K-Ras^G12C^ (right) cells that were treated with doxycycline (Dox) for 24 h to induce N-Ras^G12C^, N-Ras^G12D^, K-Ras^G12C^ or K-Ras^G12D^ expression were exposed to a range of ABD778 and sotorasib doses. **b,** pERK levels were measured by phospho-flow cytometry in MOLM13/N-Ras^G12C^ (left) and MOLM13/K-Ras^G12C^ (right) cells that were exposed to the ABD778 and/or sotorasib doses shown for 4h (n = 6–14). **c,** Bliss independence analysis of MOLM-13/N-Ras^G12D^ and MOLM13/K-Ras^G12D^ cells that were treated with a range of ABD778 and gilteritinib concentrations was performed as described above. **d,** pERK levels were measured by phospho-flow cytometry in MOLM13/N-Ras^G12D^ (left) and MOLM13/K-Ras^G12D^ (right) cells that were exposed to the ABD778 and/or gilteritinib doses shown for 4h (n = 6–7). **e,** NSG mice were transplanted with 1 million human AML PDX cells. After confirming the presence of human CD34+ cells on day 4 post-transplant, recipient mice were treated daily with vehicle, ABD778, gilteritinib, or ABD778 + gilteritinib combination (n = 6 mice per group). Mice in all four cohorts were euthanized in days 21–22 due to weight loss in mice assigned to ABD778 treatment. **f,** The percentage of human hematopoietic cells in the spleens and bone marrows of NSG mice were determined at the end of the trial by flow cytometry using an antibody to human CD45. **** p < 0.0001, ** p < 0.001, * p < 0.05.

Sotorasib is a K-Ras^G12C^ inhibitor that is also active in cancer cell lines harboring *NRAS*^*G12C*^ mutations^10^. As expected^32,51^, sotorasib inhibited the growth of isogenic MOLM-13 cells expressing either N-Ras^G12C^ or K-Ras^G12C^. ABD778 and sotorasib had synergistic anti-proliferative activity that was selective for MOLM-13 cells expressing N-Ras^G12C^ and was associated with reduced pERK levels (Figs. 6a, 6b).

Gilteritinib is approved by the FDA for patients with relapsed/refractory *FLT3*-mutant AML and is now used in many front-line treatment protocols^11,52^. In patients who enter remission, relapse after gilteritinib treatment is frequently characterized by out-growth of resistant clones harboring both the *FLT3* mutation identified at diagnosis and an oncogenic *NRAS* mutation^14^. To model this clinical situation *in vitro*, we exposed MOLM-13/N-Ras^G12D^ and MOLM-13/K-Ras^G12D^ cells to a range of gilteritinib concentrations with or without doxycycline. As expected^14^, doxycycline treatment induced gilteritinib resistance in MOLM-13/N-Ras^G12D^ and MOLM-13/K-Ras^G12D^ cells (Extended Data Fig. 11). Co-treatment with ABD778 selectively resensitized MOLM-13/N-Ras^G12D^ cells to gilteritinib as assessed by Bliss Independence analysis, which correlated with reduced pERK levels (Figs. 6c, 6d).

We next injected an aggressive PDX isolated from an AML patient with a *FLT3*-*ITD* mutation who relapsed after gilteritinib treatment with a secondary *NRAS*^*Q61K*^ mutation into NSG mice and treated them with vehicle, ABD778, gilteritinib, or a combination of both drugs. This trial was terminated after 3 weeks due to morbidity and weight loss in mice assigned to ABD778 treatment (Fig. 6e). At euthanasia, the spleens and bone marrows of mice in the vehicle group were extensively infiltrated with human CD45^+^ AML cells (Fig. 6f). ABD778 treatment modestly reduced leukemia growth in both organs, with gilteritinib showing more potent activity. However, no human cells were detected in the spleens of mice that received both drugs, and combination treatment almost eliminated PDX cells from the bone marrow (Fig. 6f).

## DISCUSSION

Oncogenic Ras proteins were historically viewed as “undruggable” due to their structural features, low nanomolar affinity for guanine nucleotides, and high intracellular GTP levels^53^. The landmark discovery and subsequent clinical development of covalent K-Ras^G12C^ inhibitors has radically changed this view, and multiple isoform-selective and pan-Ras inhibitors are either FDA-approved or undergoing clinical testing^10,54,55^. Similarly, the disappointing efficacy of farnesyl transferase inhibitors led many in academia and biopharma to abandon Ras post-translational processing as a viable target for drug discovery. Our data demonstrating the efficacy of palmitoylation cycle inhibition in AML cells harboring oncogenic *NRAS/Nras* mutations thus opens a potential new avenue for treating *NRAS* mutant cancers.

Mechanistically, we show that ABD778 potently inhibits ABHD17 enzymes, reduces N-Ras depalmitoylation, and suppresses constitutively elevated pERK levels in *NRAS*-mutant leukemia cells. Consistent with these biochemical effects, ABD778 reduced the growth of AML cell lines and JMML patient samples in a genotype-selective manner and exhibited synergistic *in vitro* and *in vivo* growth inhibition when combined with the allosteric MEK inhibitor PD901. The types of by-pass mutations detected in murine *Nras*^*G12D*^ AMLs identified after ABD778/PD901 treatment are similar to those seen in patients with lung adenocarcinomas that progressed after an initial response to a K-Ras^G12C^ inhibitor^56^ and point to the rewiring of Ras pathway activity as a more common resistance mechanism compared to the mutational disruption of drug binding to primary targets (e.g., ABHD17 enzymes, MEK). The *Kras*^*A146T*^ mutation is notable because A146T is less activating that G12D in myeloid lineage cells and other tissues^47^, yet emerged after treatment with ABD778 and PD901. Coupled with the potent *in vivo* anti-leukemia effects of combination treatment, these data support a mechanism whereby ABD778 enhances MAPK pathway inhibition and thereby shapes the genetic mechanisms deployed by primary AMLs to restore oncogenic signaling. We speculate that the general principle of enhancing therapeutic index by treating cancer cells with a potent but non-selective inhibitor of a key oncogenic driver pathway with a less-potent but highly selective compound may be applicable to targets in addition to oncogenic N-Ras.

Our data support investigating ABD778 and other ABHD17 inhibitors in combination with compounds that directly inhibit Ras^55^ or block key effectors such as Raf^57^ in cancers harboring oncogenic *NRAS* driver mutations. A combinatorial “on target” therapeutic approach may be required to increase the efficacy of chemical inhibitors of G12D and other common oncogenic amino acid substitutions that lack a “covalent handle” (i.e. an oncogenic, nucleophilic cysteine residue). Similarly, our data in a PDX model of adaptive resistance to a clinical FLT3 inhibitor raise the unexpected possibility of co-targeting N-Ras to enhance the efficacy of gilteritinib by preventing the emergence of resistance.

In conclusion, our studies validating N-Ras post-translational processing as a therapeutic target in cancer should stimulate future studies of the N-Ras palmitoylation cycle. For example, our findings indicate that the sub-chronic inhibition of ABHD17 enzymes is well-tolerated in mice, suggesting that potential effects on additional palmitoylation substrates of these enzymes^5^ do not lead to severe health impairments. Nonetheless, how ABHD17 inhibitors impact the palmitoylation and function of other dynamically palmitoylated proteins^58–63^ warrants further investigation. Additionally, the basis of the profound inhibition of MAPK signaling in response to combined ABD778/PD901 treatment is a provocative question for future studies. Inhibiting PAT enzymes or interacting proteins such as the RAB27B GTPase are possible alternative therapeutic strategies for treating *NRAS*-mutant cancers that are supported by genetic, biochemical, and phenotypic data^3,6,26,64^. From a translational perspective, we show that ABHD17 inhibition is a promising new therapeutic strategy for patients with AML and other *NRAS*-mutant malignancies such as thyroid and colorectal cancers, melanoma, and embryonal rhabdomyosarcoma.

## Methods

### ABD778 synthesis.

The Extended Data sections includes a description of the synthesis and characterization of ABD778 with the structures of intermediate compounds.

### Caco-2 permeability assay.

Compound permeability was determined using a Caco-2 cell monolayer bidirectional transport assay coupled to LC-MS/MS analysis. Caco-2 cells (50 μL at 6.86×10^5^ cells/mL) were dispensed into each well of a 96-well HTS Transwell plate and cultured for 14–18 days.

Test compound solutions were prepared as 1.0 mM stock solutions in DMSO and diluted in HBSS buffer (10 mM HEPES, 2% BSA, pH 7.4) to 5.0 μM. To measure apical-to-basolateral (A→B) transport, 75 μL of test compound solutions were added to the Transwell insert (apical compartment) and 235 μL HBSS buffer (10 mM HEPES, 2% BSA, pH 7.4) to the receiver plate (basolateral compartment). For basolateral-to-apical (B→A) transport, 235 μL of test compound solutions were added to the receiver plate (basolateral compartment) and 75 μL of HBSS buffer was added to the Transwell insert (apical compartment). Plates were incubated at 37°C for 2 h. Samples (50 μL) were collected from apical and basolateral compartments and quenched with 4 volumes of cold methanol. Samples were vortexed and centrifuged at 3,220g for 40 min, and supernatants (100 uL) were diluted 1:1 with water for LC-MS/MS analysis.

Sample extracts (3 μL) were injected onto a Waters XSelect HSS T3 C18 column (2.1 × 50 mm, 2.5 μm particle size) coupled to a Waters XSelect HSS T3 C18 guard column (2.1 × 30 mm, 2.5 μm particle size). Mobile phase A consisted of 0.1% formic acid in water, and mobile phase B consisted of 0.1% formic acid in acetonitrile. The gradient started at 5% B for 0.8 min, increased linearly to 100% B over 0.3 min, maintained at 100% B for 0.1 min, before returning to initial conditions. Flow rate was maintained at 650 μL/min.

Analytes were quantified using an API 5500 mass spectrometer equipped with an ESI interface operated in MRM mode. Source parameters were maintained as follows: collision gas = 6 L/min, curtain gas = 30 L/min, nebulizer gas = 50 L/min, auxiliary gas = 50 L/min, temperature = 500°C, and ion spray voltage = +5500V. The quantitative transition for ABD778 was m/z 669.94→369.21 (DP = 120V, EP = 8V, CE = 40V, CXP = 15V). The quantitative transition for ABD957 was m/z 628.50→327.30 (DP = 80V, EP = 8V, CE = 32V, CXP = 15V).

Apparent permeability coefficients (Papp) were calculated using the equation:

$$P_{\text{app}}=\frac{V_{A}}{\text{Area}\times \text{time}}\times \frac{{\left[\text{compound}\right]}_{\text{acceptor}}}{{\left[\text{compound}\right]}_{\text{initial}\;\text{donor}}}$$

*V*_*A*_ is the acceptor volume, Area is the membrane surface area (0.143 cm^2^), and time is the transport duration in seconds. The efflux ratio was determined using the following equation:

$$\text{Efflux}\;\text{Ratio}=\frac{P_{\text{app}(B-A)}}{P_{\text{app}(A-B)}}$$

### Cell lines and tissue culture.

OCI-AML3 (DSMZ, ACC-582), NB-4 (DSMZ, ACC-207), HL60 (DSMZ, ACC-3), SKM1 (DSMZ, ACC-547) cells, and lab-generated MOLM13 lines were grown in RPMI supplemented with 10% fetal bovine serum (FBS), L-glutamine (2 mM), penicillin (100 U ml^−1^), streptomycin (100 μg ml^−1^) and grown at densities between 0.3 × 10^6^ and 2 × 10^6^ cells mL^−1^. HEK293T (ATCC, CRL-3216) were grown in DMEM supplemented with 10% FBS, l-glutamine (2 mM), penicillin (100 U ml−1) and streptomycin (100 μg ml−1). All cells were maintained at 37 °C with 5% CO2.

### Proteome preparation.

Tissue proteomes (mouse brain, kidney, and spleen) were prepared by adding each tissue, a 5 mm stainless steel bead, and cold PBS (1.0 mL) to Safe-Lock tubes and homogenizing using the Tissue Lyser II (Qiagen) (30 Hz/sec for 30–60 sec). For spleen and kidney samples, protease inhibitors (Pierce^™^ Protease Inhibitor Mini Tablets, EDTA-free) were added to the PBS prior to homogenization. Homogenized tissues were then clarified by centrifuging (2500g) at 4 °C and discarding the pellet. Cell proteomes were prepared by sonicating cell pellets in cold PBS. To isolate the membrane fraction of tissues or cells, cell lysates or brain homogenates were subjected to ultra-centrifugation (100,000g for 45 min at 4 °C). Supernatants were removed and membrane pellets were resuspended in PBS using probe sonication. Protein concentrations were determined using Bio-Rad DC protein assay and diluted to desired protein concentration (see below) for subsequent ABPP analysis.

### Gel-based ABPP.

Inhibitor potency (IC_50_ values and target engagement) against ABHD17 and other serine hydrolases was determined by competitive gel-based ABPP in mouse brain and hABHD17B-transfected HEK293T membrane proteomes using FP-Rh competition^1^. hABHD17B in a pCMD-SPORT6 vector (MHS1010–202726047, 4748883, Dharmacon) was recombinantly expressed in HEK293T cells.

*In vitro* potency for mABHD17 and hABHD17B was determined by treating mouse brain or hABHD17B-transfected HEK293T membrane proteomes (50 μg, 1.0 mg/mL) with ABD778 (0.001 – 10 μM) or DMSO in triplicate for 30 min at 37 °C and subsequently treating with FP-Rh (1.0 μM) for an additional 30 min at room temperature. After incubation with FP-Rh, reactions were quenched with 4X SDS-PAGE loading buffer and FP-Rh-labeled enzymes were resolved by SDS-PAGE (10% acrylamide). In-gel fluorescence was visualized using a Bio-Rad ChemiDoc^™^ XRS imager. Fluorescence is shown in gray scale. Quantification of enzyme activities was performed by densitometric analysis using ImageJ software (NIH). Integrated peak intensities were generated for the band corresponding to mABHD17 in mouse brain and hABHD17B in HEK293T proteomes. Note that the identity of the ABHD17 band detected in mouse brain ABPP gels may correspond to any one or more of the ABHD17 isoforms, therefore we have not specified which isoform is being measured. ABHD17 IC_50_ values were calculated through curve fitting semi-log-transformed data (*x*-axis) by non-linear regression with a four-parameter, sigmoidal dose response function (variable slope) in Prism software (GraphPad).

### In situ selectivity profiling by MS-ABPP.

For *in situ* treatments, OCI-AML3 cells (5 × 10^6^ cells) were added to 6-well plates at a density of 1.25 × 10^6^ cells ml^−1^. Cells were treated with DMSO or ABD778 (0.001 – 10 μM) in triplicate and incubated for 4 h. Cells were pelleted at 500g by centrifugation, washed with PBS, pelleted again and snap frozen prior to cell lysis, proteome preparation and ABPP.

### MS-based ABPP sample preparation.

For *in vitro* MS-ABPP, cell or tissue proteomes (1.0 mg/mL in 0.2 mL of PBS) were treated with inhibitor (0.001–10 mM) or DMSO for 30 min at 37 °C and subsequently labeled with FP-biotin (10 μM) for 1 h at room temperature. Proteomes from *in situ*-treated cell pellets (1.0 mg/mL in 0.2 mL of PBS) or tissues collected from mice following compound treatments (5.0 mg/mL in 0.2 mL of PBS) were directly labeled with FP-biotin as described above.

Following FP-biotin treatment, proteomes were denatured and precipitated using 9:1 acetone/MeOH, resuspended in 0.2 mL of 8 M urea in PBS and 1% SDS, reduced using DL-dithiothreitol (DTT, 10 mM) for 20 min at 55 °C, and then alkylated using iodoacetamide (50 mM) for 30 min at room temperature in the dark. The biotinylated proteins were enriched with PBS-washed streptavidin-agarose beads (50 μL; Thermo Scientific) by rotating at room temperature for 1.5 h in PBS with 0.2% SDS (1.3 mL). The beads were then washed sequentially with 0.5 mL 0.2% SDS in PBS (10×), 1.1 mL PBS (10×) and 1.1 mL DI H_2_O (10×). On-bead digestion was performed using sequencing-grade trypsin (2 μg; Promega) in 2 M urea in PBS for 12–14 h at room temperature (100 μL). The beads were removed using filtration and washed with DPBS (100 μL). Protein digests were acidified by addition of formic acid (10 μL of 100% formic acid) and desalted using SOLAμ^™^ SPE Plates (HRP 2 mg / 1 mL). Samples were dried by centrifugal evaporation and stored at −80 °C.

For *in vitro* and *in situ* ABPP samples, peptides were labeled with TMTpro reagents using protocols adapted from previous work^2^. Dried samples were reconstituted in 20 μL EPPS buffer, pH 8.5 followed by addition of 5 μL 40 mM TMTpro reagents in acetonitrile for 1 hr. Reactions were quenched with 5 μL 5% hydroxylamine, mixed, desalted, dried by centrifugal evaporation, and stored at −80 °C until analysis.

#### MS data acquisition and analysis

##### Parallel reaction monitoring (PRM) for in vivo target engagement

Dry peptide samples were reconstituted in water containing 0.1% formic acid (20 μL) and 10 μL were injected onto an EASY-Spray column (15 cm × 75 μm ID, PepMap C18, 3 μm particles, 100 Å pore size, Thermo Fisher Scientific) using a Vanquish Neo UHPLC (Thermo Fisher Scientific). Peptides were separated over a 15 min gradient of 0 to 40% acetonitrile (0.1% formic acid) and analyzed on an Orbitrap Fusion Lumos (Thermo Fisher Scientific) operated using a parallel reaction monitoring (PRM) method targeting select peptides from ABHD17A, ABHD17B, ABHD17C, LYPLA1 and LYPLA2 as well as additional control peptides to assess sample integrity: PCCA and PC. Selected ions were isolated and fragmented by high energy collision dissociation (HCD) at 30% CE and fragments were detected in the Orbitrap at 15,000 resolution. Further details for the targeted peptides can be found in Table 1.

##### MS Acquisition for TMTpro Labeled Peptides.

MS was performed using a Thermo Scientific Vanquish Neo and Orbitrap Eclipse system. Peptides were eluted over a 240 min nLC 5-25-45% acetonitrile gradient. For all samples, data were collected in data-dependent acquisition mode over a range from 375–1,500 m/z. Each full scan was followed by fragmentation events for 1.5 sec including real-time search of mouse or human peptides with TMTpro modifications and carbamidomethylation of cysteine and oxidation of methionine for subsequent synchronous precursor selection. Dynamic exclusion was enabled (repeat count of 1, exclusion duration of 60 s) for all experiments.

##### TMTpro Data Analysis.

Data analysis was performed using a custom in-house pipeline. Raw files were converted to the indexed mzML format using ThermoRawFileParser v1.4.2^3^ and then searched with Comet v2019.01.5^4^ against a human FASTA database obtained from UniProt^2^ (reference proteome UP000005640_9606, downloaded on 2020-03-21), and concatenated with a list of non-human contaminant proteins obtained from MaxQuant^5^, and reverse decoys. For the Comet search, TMTPro modifications on lysine and the N-terminus were specified as static modifications, in addition to a static modification for carbamidomethylation at cysteine [+57.021463], and a differential modification for oxidation at methionine [+15.994914]. Further processing was performed using the OpenMS platform v3.0.0^6^ (commit short hash 26292ad). Files were first converted from .pep.xml to .idXML using IDFileConverter, target/decoy information was added using PeptideIndexer, features were extracted with PSMFeatureExtractor, and Percolator v3.05^7^ was run through PercolatorAdapter. Peptide spectrum matches (PSMs) were then filtered with a q-value threshold of 0.01. Further processing was then carried out using custom Python code. For each filtered PSM, MS3 scans were extracted from the original mzML file, and reporter ion intensities were extracted from the most intense peak within a 0.002 Th window of each reporter ion expected mass. Intensity values were then normalized per channel to the mean per-channel summed intensity. PSMs were excluded from downstream calculations if their control sum intensity was less than 5000 x N where N is the number of channels corresponding to the control condition. Intensity values for each PSM were then summed per-channel for each protein, and percent activity values were calculated by dividing each value by the mean intensity across the summed control channels. The mean was then taken across channels associated with the same condition and was reported as a percent activity value for each protein.

### *In vivo* target engagement and bioanalysis of ABD778 and ABD957.

Female C57Bl/6J mice (Jackson) aged 10 weeks at the time of dosing were administered ABD778, ABD957 or PD-901 by oral gavage (10 ml/kg volume). Each compound was prepared fresh on the day of dosing in PEG400:ethanol:PBS (7:2:1) for ABD778 and ABD957 or 0.6% methylcellulose and 0.2% Tween80 in water for PD901. Maximal dispersal of the compound was achieved by bath and probe sonication until a uniform white suspension was formed. For combination studies, PD901 was administered 1 h prior to ABD778. Animals were anesthetized with isoflurane at the indicated time points after compound administration. Blood was collected by cardiac puncture into EDTA microtubes, quenched with 4 volumes of cold acetonitrile and centrifuged at 17,000 x g for 2 minutes prior to collecting the supernatants for analysis. Spleen and kidneys were then removed and rinsed in PBS before freezing in liquid nitrogen. Tissues were stored at −80 C until analysis by ABPP (see below). For pharmacokinetic analysis of ABD778 and ABD957-derived from hydrolysis of ABD778, blood (~30 μL) was serially collected from the dorsal metatarsal vein and quenched with cold acetonitrile (120 μL) and stored at −80 C until analysis.

### LC/MS analysis of compound concentrations from whole blood.

Compound concentration in acetonitrile-quenched whole blood was determined by ultra-performance liquid chromatography coupled to mass spectrometry (UPLC/MS). The calibration curve samples were prepared using whole blood from naïve mice quenched with 4 volumes of acetonitrile following the same protocol as above and spiked with serial dilutions of the test article, ranging 0.1–5000 ng/mL. Samples were incubated on ice for 60 minutes and then centrifuged at 2,400 *g* at 4 °C for 15 minutes and supernatants were transferred to another tube. 20 mL of supernatant were mixed with 80 mL of water and transferred to a LC vial containing a borosilicated glass insert for UPLC/MS analysis.

Whole blood extracts (10 mL) were injected onto an Agilent 1290 UPLC system equipped with a G7120A pump, a G7167B multisampler and a G1170A column manager (Agilent Technologies, Santa Clara, CA). Chromatographic separation was achieved using an Acquity UPLC BEH C18 Column (2.1 × 50 mm, 1.7 mm particle size, 130 Ǻ) coupled to an Acquity UPLC BEH C18 Column Guard (2.1 × 5 mm, 1.7 mm particle size, 130 Ǻ) (Waters Corporation, Milford, MA). Mobile phase A was composed of water/acetonitrile 95:5 (v/v) and mobile phase B was composed of acetonitrile/water 95:5 (v/v) with 0.1% formic acid added to both mobile phases. The gradient started with 0% B for 0.5 min before being increased linearly to 100% B over 4 min. Afterwards, solvent B was kept at 100% for 1 min, before switching to the initial conditions in 0.1 min. The system was allowed to equilibrate for 1.4 minutes before the next sample injection. Flow rate was kept at 0.6 mL/min and the column temperature was 50 °C.

Analytes were quantified using a 6470 triple quadrupole mass spectrometer equipped with an electrospray Jet Stream source (Agilent Technologies) operated in dynamic multiple reaction monitoring (dMRM) mode. The quantitative and qualitative transitions for each compound were optimized using the authentic standards in the Optimizer software (Agilent Technologies): ABD778 (Quant: 670.3→369.1, CE=22 eV; Qual: 670.3→ 240.0, CE= 45 eV); PD901 (Quant: 483.→249.0, CE= 22 eV; Qual: 483.0→ 375.9, CE= 10 eV). The following parameters were kept constant for all transitions: Fragmentor=180, Cell Accelerator Voltage=4, Polarity=Positive. Total cycle time was 500 ms. Source parameters were kept as follows: Dry Gas Temperature=350 °C, Dry Gas Flow=11 L/min, Sheath Gas Temperature=350 °C, Sheath Gas Flow=11 L/min, Nebulizer= 50 psi, Noozle voltage= 1500 V (positive) and Capillary= 3500 V (positive). Compounds concentrations were calculated by interpolating the integrated area under the curve with the calibration curves for each tissue prepared in the same matrix than the samples using the Masshunter Quantitative Analysis Software (Agilent Technologies).

### Isogenic OCI-AML3/ON and ONK cell lines,

A pCDH-LMN–GFP lentiviral vector was obtained by cloning the miR30-PGK-NeoR-IRES–GFP cassette from LMN–GFP^8^ into a pCDH Expression Lentivector (System Biosciences). A miR30-based shRNA targeting human *NRAS* (sense, 5′-CAGGGTGTTGAAGATGCTTTT-3′) was cloned into the vector. The coding sequence of *Nras*^*G12D*^ was cloned downstream of GFP to create a N-terminal GFP-fused N-Ras^G12D^ expression construct with N-Ras endogenous HVR (N-HVR). Alternatively, a chimeric version was cloned where the sequence corresponding to amino acids 166–188 from N-Ras was replaced with that of K-Ras4b (K-HVR). Lentiviral vector production and transduction of OCI-AML3 cells were performed as previously described^1^.

### Dynamic palmitoylation assay.

Dynamic palmitoylation assay was performed as described previously^1^. In brief, OCI-AML3 cells were grown as described above, then spun down (3 minutes, 500*g*) and resuspended in fresh medium at a density of 2 × 10^6^ cells ml^−1^ (10 × 10^6^ cells per sample for gel-based assay and 20 × 10^6^ cells per sample for MS-based assay). Cells were preincubated with inhibitor or DMSO for 1 h, then 17-ODYA (20 μM) was added for 1 h. Samples were pelleted and snap frozen immediately following the 17-ODYA ‘pulse’ and designated *t*_0_, or resuspended in pre-warmed chase medium which consisted of OCI-AML3 growth media, supplemented with DMSO or inhibitor at the same concentration as in the preincubation step and incubated for 1 h. Cells were then centrifuged, placed on ice, washed in cold PBS, snap frozen and designated *t*_1_.

### Immunoprecipitation.

Cell pellets were resuspended in cold lysis buffer which consisted of 1% Triton-X PBS with 1 mM phenylmethyl sulfonyl fluoride (PMSF), 0.2 mM hexadecyl sulfonyl fluoride (HDSF), 20 μM HDFP and protease inhibitors (complete Ultra EDTA-free mini tablets, 5892791001 Roche), sonicated with a microtip probe sonicator (seven times, 50% rate, power 4), and placed on an end-over-end rotator for 30 minutes at 4°C. Samples were then hard spun (16,300*g*, 5 minutes) and the supernatant was transferred to fresh tubes on ice. Protein concentration was measured using a detergent compatible (DC) assay kit from Bio-Rad and adjusted to 1 mg ml^−1^. Input samples were taken at this point and stored at −80°C.

Enrichment was performed with anti-GFP Sepharose beads (20 μl of 50:50 slurry, ab69314, Abcam; RRID:AB_1640178). Antibody conjugated beads were spun down (500*g*), storage buffer was aspirated with a 26-gauge needle, and beads were washed three times with 1% Triton-X PBS. To each sample, 20 μl of washed bead slurry was added using a cut pipette tip, and samples were placed on an end-over-end rotator for 3 hours at 4°C. Next, washes were performed by centrifugation/aspiration (3×, 500*g*) with cold 1% Triton-X PBS containing 500 mM NaCl. After the last wash, supernatant was removed, and a 26-gauge needle was quickly inserted into the bead slurry to remove all remaining liquid. GFP beads were then resuspended in 50 μl of wash buffer. At this point, immunoprecipation samples were either stored at −80°C or processed further for analysis by SDS–PAGE gel.

### Click chemistry and processing for SDS–PAGE gel.

On-bead click chemistry was performed to conjugate a rhodamine fluorophore reporter to 17-ODYA labeled proteins. To each 50 μl sample, 6 μl of a click chemistry reaction mixture was added. The click reaction mixture was freshly prepared as follows (amounts given are per sample): 1 μl of 50 mM CuSO_4_ (in water; final concentration during reaction of 1 mM), 3 μl of 1.7 mM tris(benzyltriazolylmethyl)amine (4:1 *t*-BuOH/DMSO; 100 μM final), 1 μl of 50 mM TCEP (freshly made in PBS; 1 mM final) and 1 μl of Rh-N_3_ (DMSO; 25 μM final). Reactions were allowed to proceed for 1 hour at room temperature. Samples were then quenched with 4× SDS loading buffer containing 1% β-mercaptoethanol (described below), boiled for 5 minutes to effect elution and finally 17-ODYA proteins were resolved and imaged on gel.

Loading buffer was prepared as follows (for 100 ml): 3.02 g of Tris base was added to 40 ml of water, 40 ml of glycerol was added slowly and the mixture was stirred using a stir bar while the pH was brought to 6.75 using concentrated HCl; 8 g of SDS were then added followed by 20 ml of water and a pinch of bromophenol blue. Loading buffer was stored at room temperature, and 1% (10 μl per 1 ml of buffer) β-mercaptoethanol was added immediately before quenching.

Finally, either 15 μg protein samples were loaded on to a 4–20% Criterion^™^ TGX gels (Bio-Rad) resolved by SDS–PAGE (160 V for ~1 hour) or 30 μg protein samples were loaded on to a 10% acrylamide gel, resolved by SDS–PAGE (300 V for ~2.5 hours), and in-gel fluorescence was visualized using a Bio-Rad ChemiDoc MP flatbed fluorescence scanner.

### Cell viability analysis.

Cell lines were plated at 1,000 cells/well on white opaque 96-well flat bottom plates (Perkin Elmer) in technical triplicate. Cells were treated with indicated drug dose series for 72 hours. Metabolically active cells as a proxy for overall cell viability were measured with CellTiter-Glo (Promega) and analyzed on a plate reader (Tecan M2000 Infinite Pro). Experiments were performed with technical triplicates and a representative experiment is shown from at least three experimental replicates for each cell line.

### Phospho-flow cytometry.

Cells were incubated with drug and/or vehicle for 4 hours, then were fixed with a final concentration of 2% paraformaldehyde (Electron Microscopy Sciences). Samples were permeabilized with ice-cold methanol, washed, then stained with pERK (Cell Signaling Cat#9101, RRID:AB_331646) or pS6 (Cell Signaling Cat#4858, RRID:AB_916156). Cells were then washed again, stained with a secondary antibody (Jackson Immunoresearch Cat#711-135-152, RRID:AB_2340601), and analyzed on a LSR II flow cytometer (BD Biosciences).

### Immunoblotting.

Cells were incubated with drug and/or vehicle for 4 hours, then were lysed in ice cold RIPA buffer (Pierce) supplemented with HaltTM protease and phosphatase inhibitor cocktail (Thermo Fisher) and 0.5 μM EDTA, incubated on ice for 30 minutes, and centrifuged at maximum speed for 15 minutes to collect whole cell lysates. Protein concentration was measured with the BCA protein assay (Pierce). 30μg of total protein per sample was loaded into 4–12% gradient Criterion^™^ TGXTM gels (Bio-Rad) and separated by SDS-PAGE. Proteins were transferred to PVDF or nitrocellulose membranes and blotted with the following primary antibodies: pERK (Cell Signaling Cat#9101, RRID:AB_331646), ERK (Cell Signaling Cat#9107, RRID:AB_10695739), pS6 (Cell Signaling Cat#4858, RRID:AB_916156), S6 (Cell Signaling Cat#2317, RRID:AB_2238583), pAKT (Cell Signaling Cat#4060, RRID:AB_2315049), AKT (Cell Signaling Cat#2920, RRID:AB_1147620). GAPDH (Cell Signaling Cat#97166, RRID:AB_2756824), Ras^G12D^ (Cell Signaling Cat#14429, RRID:AB_2728748), and Hsp90 (Cell Signaling Cat#4875, RRID:AB_2233331) was used for loading controls.

### Colony forming assay.

Incomplete MethoCult^™^ (STEMCELL Technologies) was supplemented with IMDM +/− GM-CSF and ABD778 (final concentration 1μM) or vehicle per the manufacturer’s recommended protocol. Cryopreserved patient JMML bone marrow cells were mixed with supplemented MethoCult^™^ to achieve a final concentration of 2750 CD34^+^ cells / 3.3mL media. 1.2mL of cell / MethoCult^™^ mixture (containing 1000 CD34^+^ cells) were aliquoted in duplicate into 30mm plates, and then incubated for 14 days at 37 °C with 5% CO2 prior to colony counting.

### Preclinical trials in mouse AMLs.

Transplantable primary AMLs were generated by injecting neonatal wild-type (WT), *Mx1-Cre; Nras*^*LSL-G12D/+*,^ or *Mx1-Cre; Kras*^*LSL-G12D/+*,^ mutant mice with the MOL4070LTR retrovirus followed by a single dose of polyI-polyC at weaning to induce *Nras*^*G12D*^ or *Kras*^*G12D*^ expression from the endogenous loci as described (see Refs 37, 41). Preclinical trials were performed by expanding cryopreserved leukemia cells *in vivo* and transplanting 1 ×10^6^ bone marrow cells into irradiated (450cGy) 8- to 12-week-old congenic recipients. Mice were dosed daily with ABD778 (60 mg/kg), PD901 (2.5 mg/kg), this combination, or vehicle by oral gavage. Drug and vehicle were made fresh each week. Mice were monitored daily until they became symptomatic, then twice daily until moribund. Bone marrow, spleen, and cardiac blood was collected for genomic DNA extraction, cryopreservation, and complete blood count analysis (HemaTrue).

### Whole exome sequencing.

Genomic DNA was extracted from the bone marrow cells and then sheared to generate 150 to 200 base pair fragments using a Covaris S2 focused-ultrasonicator. Indexed libraries were prepared using the Agilent SureSelect XT2 Reagent Kit for the HiSeq platform. Exomes were captured using the Agilent SureSelect XT2 Mouse All Exon bait library. Sample quality and quantity were assessed using the Agilent 2100 Bioanalyzer instrument. Paired-end 100 base pair reads were generated on an Illumina HiSeq 2000 platform. All sequence data including read alignment; quality and performance metrics; post-processing, somatic mutation and DNA copy number alteration detection; and variant annotation were performed as previously described^9^ using the mm10 build of the mouse genome. Briefly, reads were aligned with Burrows-Wheeler Aligner, and processed using Picard (http://broadinstitute.github.io/picard) tools and the Genome Analysis Toolkit (GATK) to perform base quality recalibration and multiple sequence realignment. Single nucleotide variants and indels were detected with the MuTect and Pindel algorithms, respectively. Candidate somatic mutations were manually reviewed using Integrative Genomics Viewer.

### Plasmids, cloning, and mutagenesis.

The plasmids pCW57.1 (Addgene plasmid 41393) and pTwist-ENTR BRAF^G466E^-T2A-mCherry (custom order, Twist Biosciences) were used to generate a dox-inducible pCW57.1-BRAF^G466E^-mCherry construct with Gateway LR Clonase enzyme mix (Thermo Fisher). The dox-inducible pCW57.1-mCherry-Kras^A146T^ vector had been previously generated by similar methods (see Ref 31).

### Lentiviral generation and transduction.

Lentiviral backbone, pMD2.G (Addgene plasmid #12259) and psPAX2 (Addgene plasmid #12260) were transfected into 293T lenti-X cells (Takara Bio) with TransIT-LT1 (Mirus Bio). Supernatant was collected 48 hours after transfection and applied to OCI-AML3 cells with polybrene for transduction. Cells were spin-infected at 800*g* for 2 hours at 37°C. After recover, cells were treated with 2 μg/mL doxycycline (Sigma) and transduction efficiency was determined by BD LSR II analysis. MOLM13 lines were previously generated by similar methods (see Ref 31).

### Competitive growth assay.

Transduced OCI-AML3 cells were grown in the presence of 2 μg/mL doxycycline and drug or vehicle as indicated. Cell viability (Beckman Coulter Vi-Cell XR) and fluorescent protein expression (BD LSRII) were measured every 2 days for 10 total days. After measurements, cells were re-plated with fresh doxycycline and drug or vehicle at a density of 0.5 × 10^6^ cells / well.

### Patient-derived xenograft trial.

NOD.Cg-Prkdc^scid^ Il2rg^tm1Wjl^/SzJ (NSG) were obtained from in-house breeding stocks at the UCSF Preclinical Therapeutics Core facility housed and bred in a pathogen-free facility. A mixture of male and female mice between 6 and 12 weeks old were transplanted with 1e6 viably frozen passaged PDX spleen cells via tail vein injection after two consecutive days of busulfan conditioning (25 mg/kg) and one day of rest. Tumor engraftment was assessed by flow cytometry for hCD45^+^ percentage in PB from submandibular bleeding on day 4. Mice were randomized to receive one of four treatments, six mice per treatment arm: (1) ABD778 50 mg/kg daily, (2) gilteritinib 30 mg/kg daily (C) ABD778 50 mg/kg daily and gilteritinib 30 mg/kg daily or (4) vehicle. Mice were analyzed for hCD45^+^ at the mid-point of the trial via submandibular bleeding. All mice were sacrificed 21 days after initiation of treatment at which point cardiac blood, spleen, and bone marrow cells were analyzed for hCD45^+^.

### Statistical Analysis.

Statistical analyses were performed using GraphPad Prism 10. Multiple comparisons between treatment conditions were performed using one way ANOVA and *Šídák* multiple comparisons testing. Kaplan Meier statistics were calculated by two-sided log rank test. In the figures, the degree of significance is denoted by the number of asterisks (****P < 0.0001, ***P ≥ 0.0001 and < 0.001, **P ≥ 0.001 and < 0.01, *P ≥ 0.01 and < 0.05, NS ≥ 0.05). Error bars indicate the mean ± s.d. unless otherwise indicated in the figure legend.

## Supplementary Material

Supplement 1

## Figures and Tables

**Table 1:** 

Protein	Peptide Sequence	m/z	Precursor Charge	RT Time (min)
ABHD17A	ELDTIEVFVTK	647.3505	2	10.5
ABHD17A	FISQELPSQR	602.8197	2	7.2
ABHD17B	WTLHLSER	521.2774	2	7.5
ABHD17B	ADWQYSSR	506.7278	2	6.2
ABHD17C	ELDAVEVFFSR	656.3326	2	11.8
ABHD17C	VAFPDTR	403.2138	2	6.5
LYPLA1	ASFSQGPINSANR	674.8338	2	6.4
LYPLA2	NFPQAANGSAK	552.7753	2	5.2
PCCA	VNTIPGFDGVVK	623.3455	2	9.2
PCCA	NFYFLEMNTR	667.8135	2	10.7
PC	SLPDLGLR	435.7558	2	8.8
PC	GTPLDTEVPLER	663.8486	2	8.7
PC	DFTATFGPLDSLNTR	827.9072	2	11.6
