# Supplementary material for: In vivo validation of the palmitoylation cycle as a therapeutic target in NRAS-mutant cancer
Source: bioRxiv. 2025 Mar 21:2025.03.20.644389. Preprint. [Version 1] doi: 10.1101/2025.03.20.644389 (PMC11957127; doi:10.1101/2025.03.20.644389)

Extended Data for Decker, *et alia:*

"In vivo validation of the palmitoylation cycle as a therapeutic target in NRAS-mutant cancer"

(1) Extended Data Figures 1-11

(2) Extended Data Tables 1-4

(3) Extended Methods: Synthesis and Characterization of ABD778

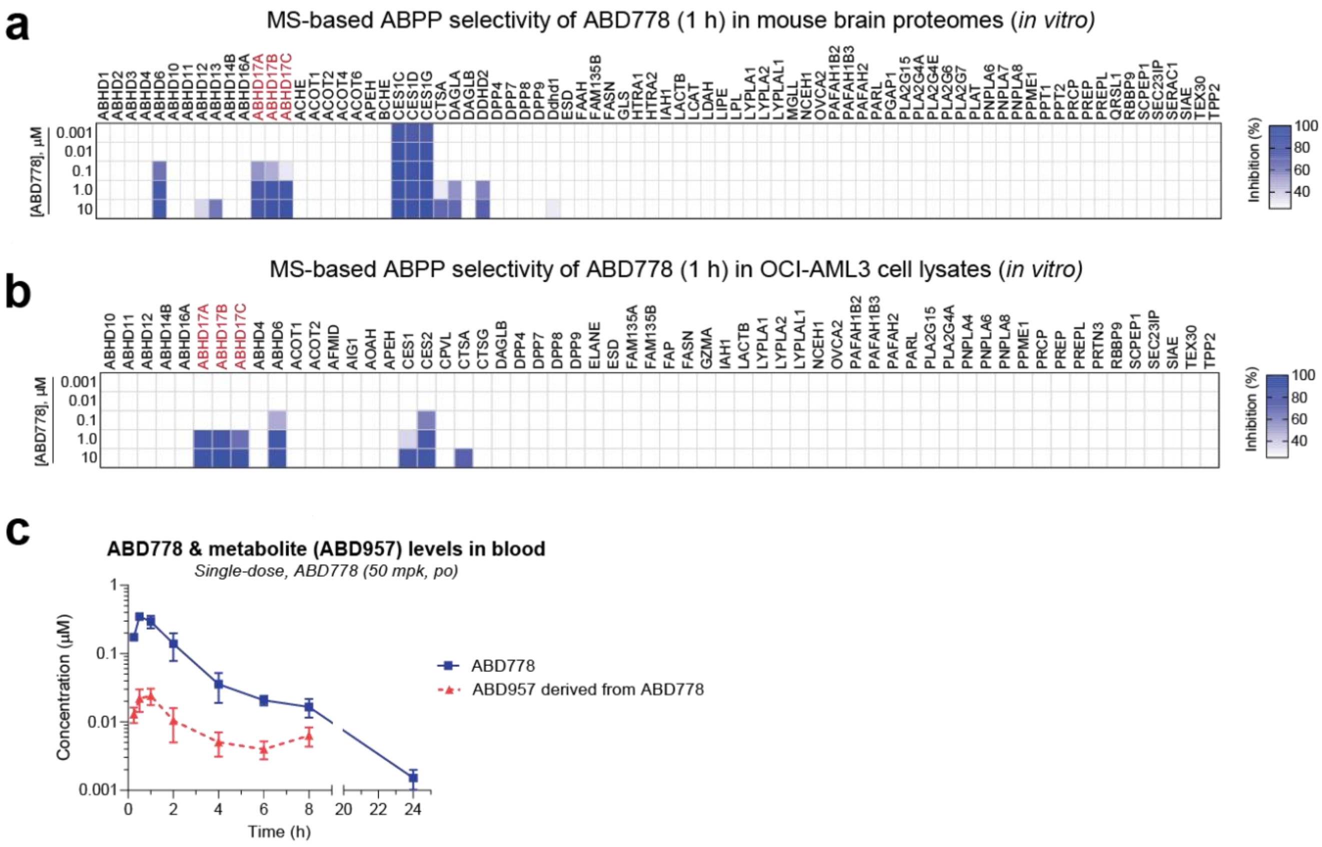

Extended Data Figure 1. Characterization of ABD778 as an ABHD17 inhibitor with *in vivo* activity. a, b, In depth serine hydrolase selectivity profiling by MS-based ABPP for ABD778 (0.001–10 µM, *in vitro*) in mouse brain (**a**) and OCI-AML3 (**b**) cell proteomes confirming ABHD17A/B/C inhibition and selectivity across the serine hydrolome including for the depalmitoylases LYPLA1, LYPLA2 and ABHD10. Prominent off-targets include ABHD6 and carboxyesterases (CESs). Data plotted represent mean competition from three biological replicates. **c,** Blood concentrations of ABD778 and ABD957 following a single dose of ABD778 (50 mg/kg, po) in mice. Blood was serially collected at the indicated timepoints following compound administration and analyzed LC-MS/MS. Note that the oncentrations of ABD957 arising from the esterolysis of ABD778 (red dotted line) are far below those of ABD778 (blue line).

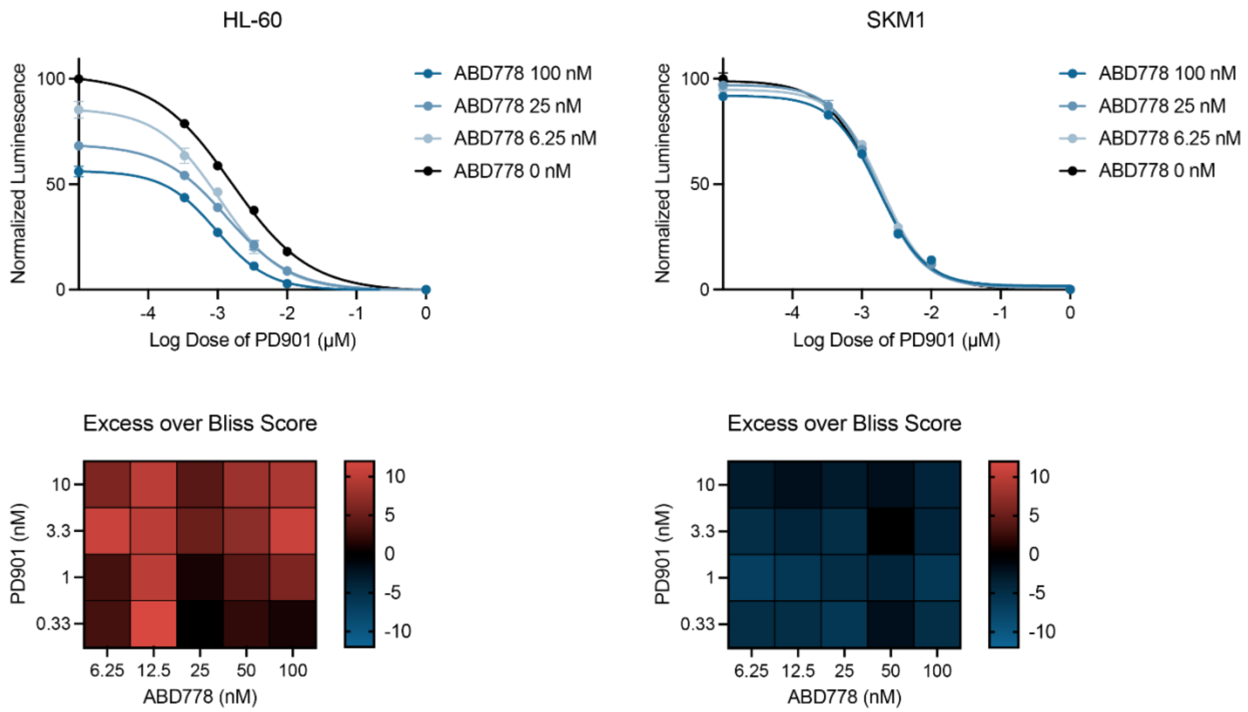

Extended Data Figure 2. Selective activity of ABD778 and synergy with PD901 in *NRAS*-mutant AML cell lines. Bliss independence analysis demonstrating synergistic growth inhibition over a broad range of ABD778 and PD901 concentrations in *NRAS*-mutant HL-60 (left), but not *KRAS*-mutant SKM1 (right), AML cells. Heatmaps display calculated synergy scores from strongly positive (red) to negative (blue). These data were generated in triplicate and were replicated in at least two additional independent experiments.

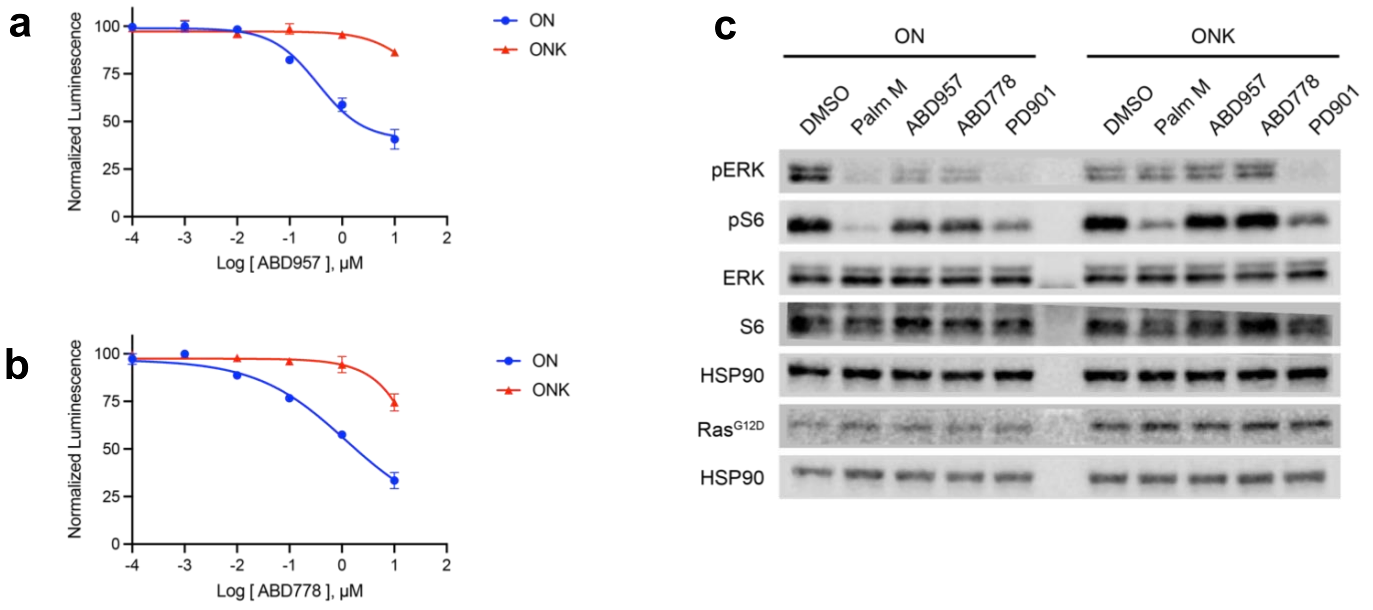

Extended Data Figure 3. a,b Cell growth as assessed by Cell Titer Glo in OCI-AML3/ON (ON) and OCI-AML3/ONK (ONK) cells exposed to a range of ABD957 (panel **a**) and ABD778 (panel **b**) doses. **c,** Western blot analysis of ON and ONK cells were exposed to either the promiscuous serine hydrolase inhibitor Palmostatin M (10 µM), ABD957 (500 nM), ABD778 (500 nM), or PD901 (10 nM) for 3h showing levels of total (t) and phosphorylated (p) ERK1/2 and S6. Antibodies to HSP90 and Ras^{G12D} were used as loading controls.

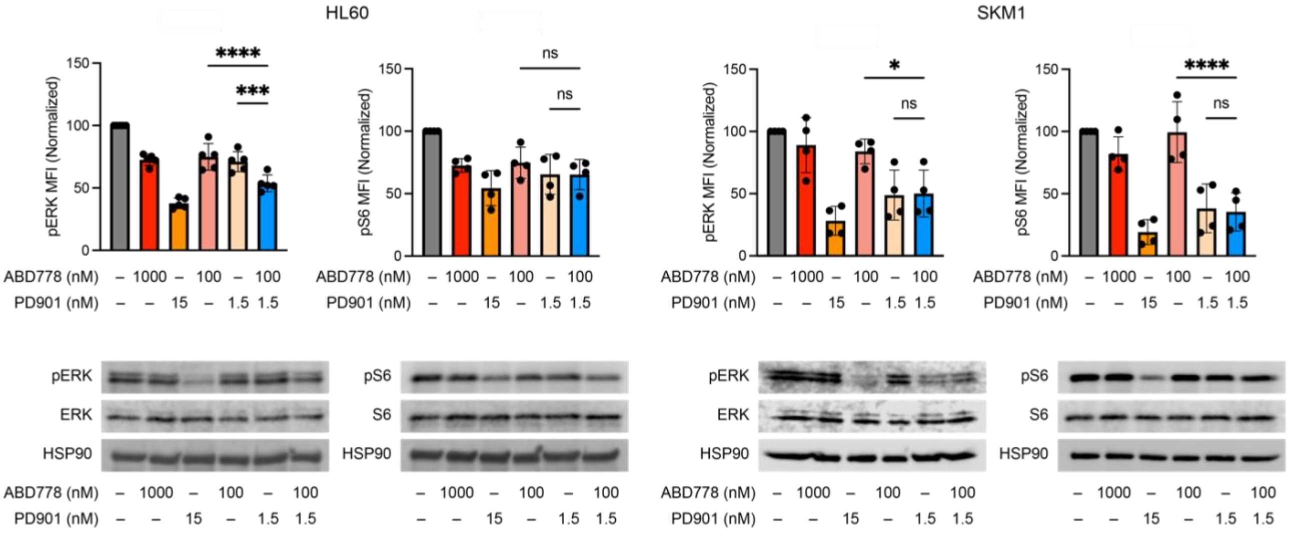

Extended Data Figure 4. Biochemical analysis of HL-60 and SKM1 AML cells that were exposed to DMSO vehicle, ABD770, PD901, or both drugs. Top**.** Phosphorylated ERK **(**pERK) and S6 (pS6) levels were measured using phospho-flow cytometry in HL-60 (left) and SKM1 (right) cells that were exposed to ABD778 and/or PD901 for 4 hours at the doses shown. Mean fluorescence index (MFI) values were normalized to 100% of the DMSO control and pooled for statistical analysis (n = 4-5; * - $p < 0.05$; *** - $p < 0.001$; **** - $p < 0.0001$). Bottom**.** pERK and pS6 levels were measured by Western blotting in HL-60 (left) and SKM1 (right) cells that were exposed to DMSO, ABD778, PD901, or both drugs. The phospho-flow were generated in three independent experiments and the Western blot data are respentative of at least three independent experiments.

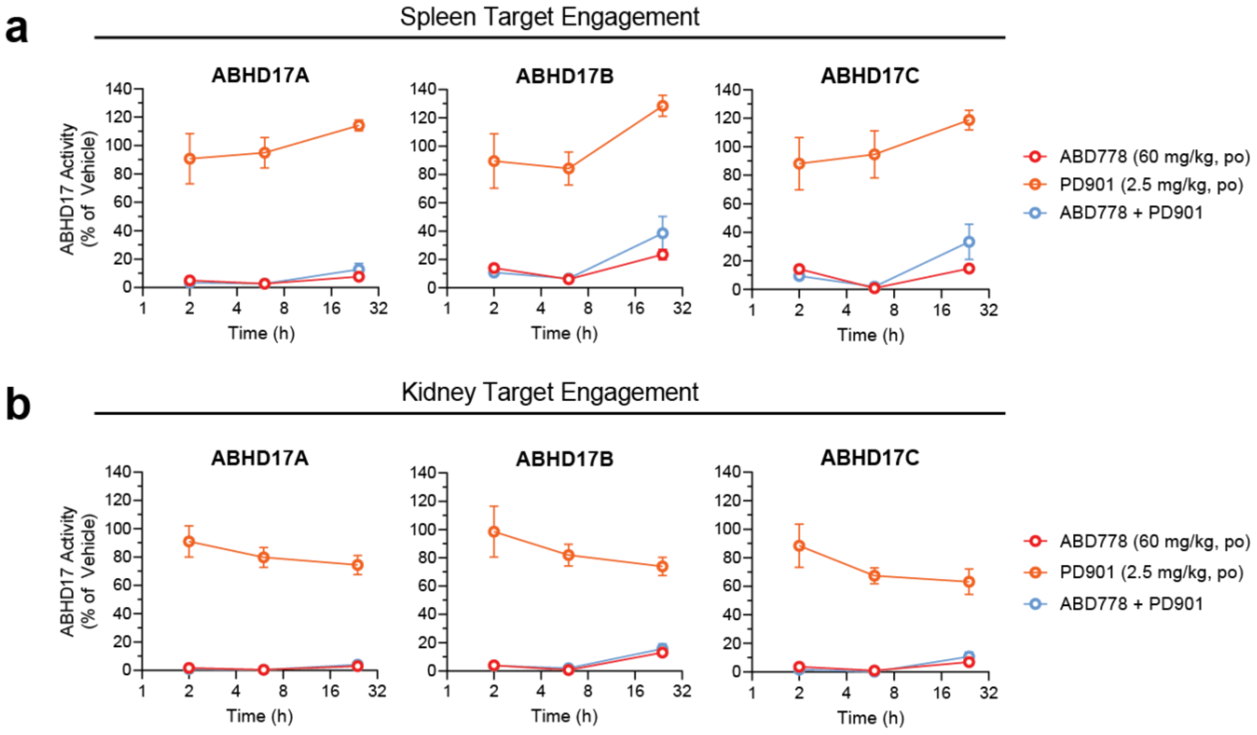

Extended Data Figure 5. Single agent and combination treatment result in similar inhibition of ABHD17 activity in hematopoietic tissues. a, **b**, *In vivo* target engagement for ABHD17A/B/C in spleen (**a**) and kidney (**b**) measured by MS-based ABPP. C57Bl/6 mice were dosed with vehicle, ABD957 (60 mg/kg, po), PD901 (2.5 mg/kg, po) or both compounds dosed 1 hour apart. Tissues were collected 2, 6, and 24 hours after compound administration and analyzed by targeted MS-ABPP using FP-biotin enrichment of serine hydrolase enzymes and parallel reaction monitoring (PRM) to detect and quantify unique diagnostic peptides from ABHD17A, B and C. ABD778 provided near complete blockade of ABHD17 enzymes in spleen and kidney across the 24 hour time course when dosed as a single agent or in combination with PD901. Data plotted represent the median from biological replicates, and error bars represent s.e.m. (n = 3-4).

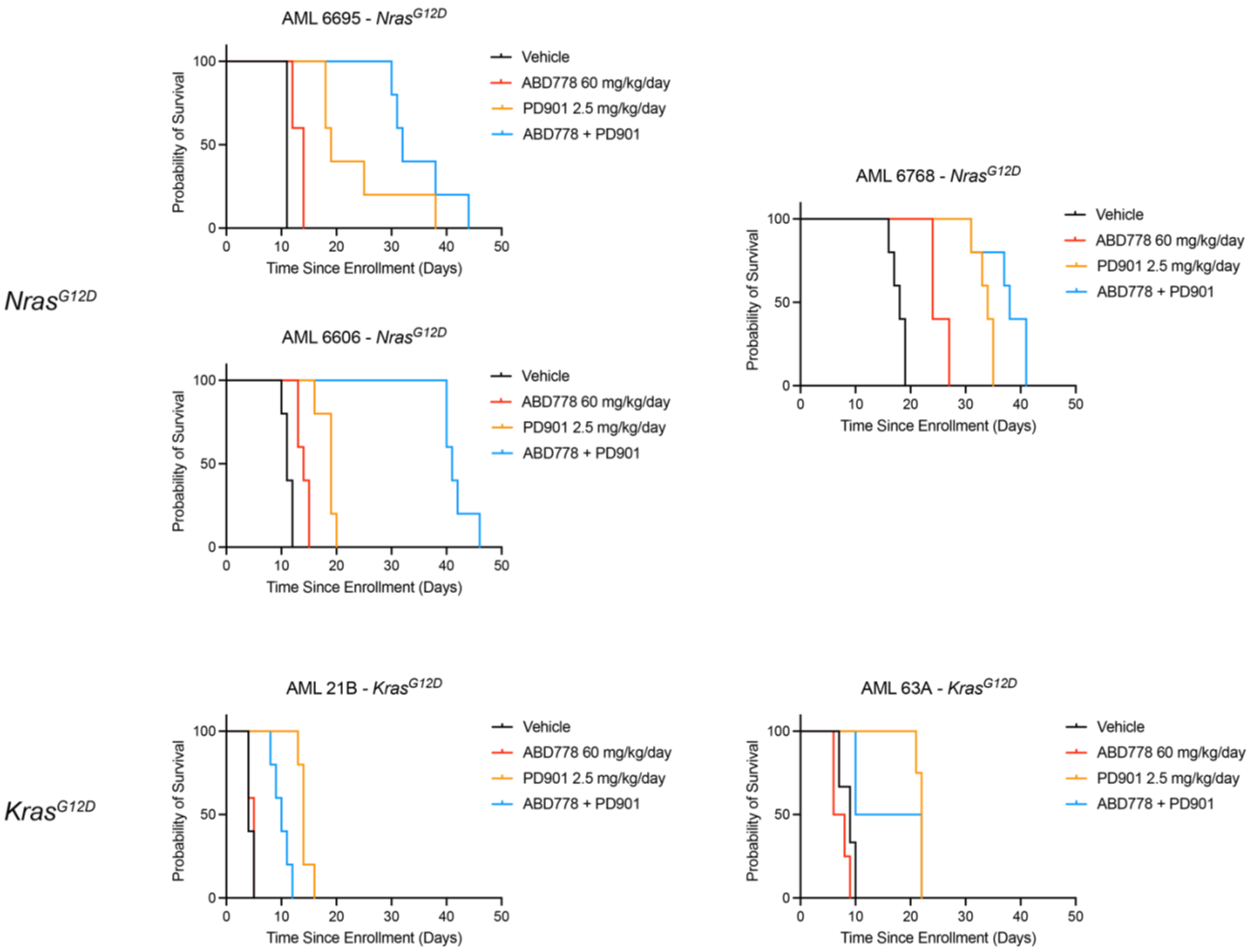

Extended Data Figure 6. Kaplan-Meier survival analysis of primary *Nras*^{G12D} (n = 3) and *Kras*^{G12D} (n = 2) AMLs enrolled on preclinical trials of ABD778 and PD901 (n = 5 mice per group x 4 groups = 20 mice in each trial).

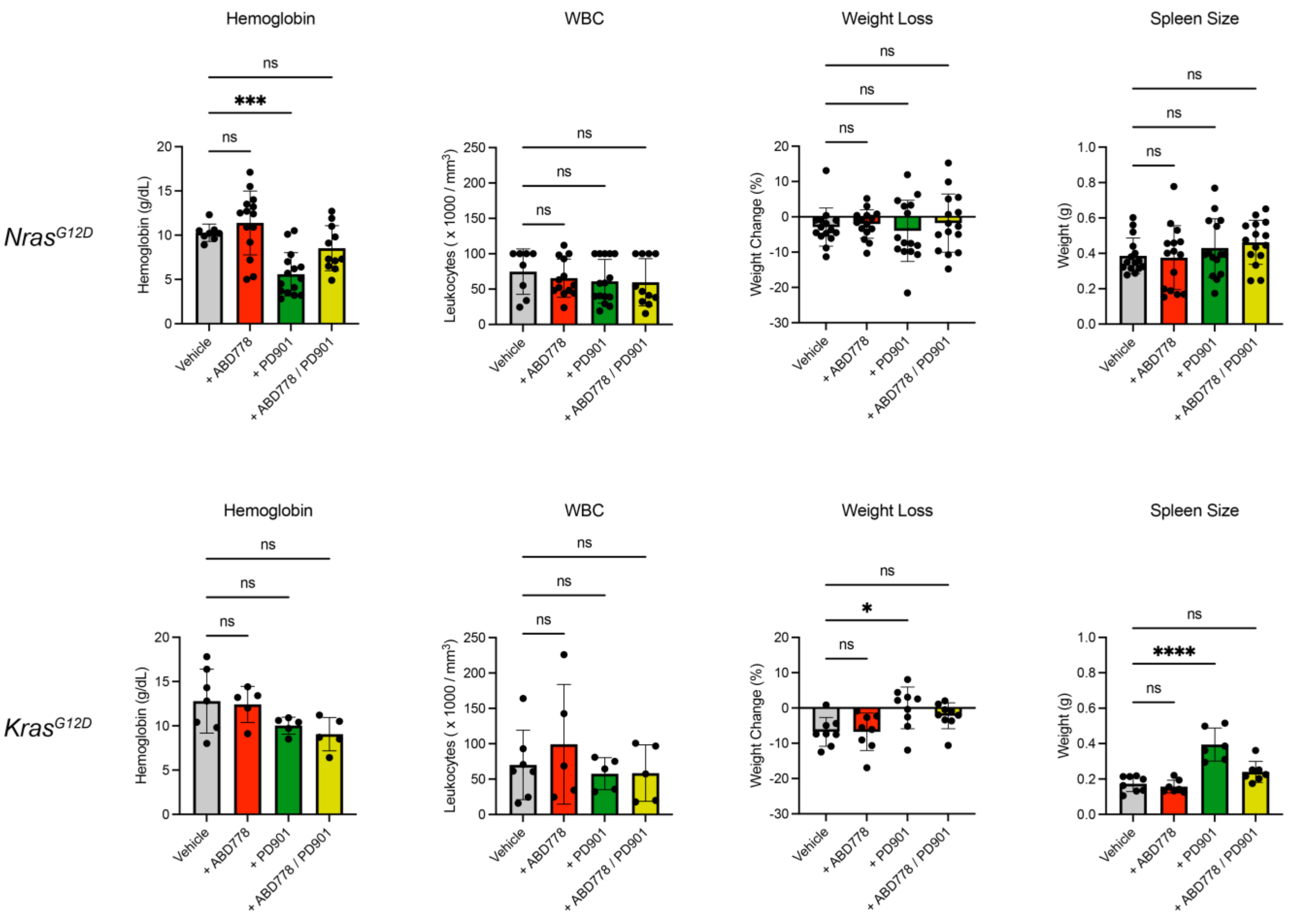

Extended Data Figure 7. Treatment-refractory AML in recipient mice at euthanasia. Despite significant differences in overall survival in some treatment arms, recipient mice in all treatment groups died with anemia (normal > 13g/dL), elevated blood leukocyte counts (normal < 14,000 per mm^3), and splenomegaly (normal < 100mg), which is indicative of refractory AML. n = 5-15; * - $p < 0.05$; *** - $p < 0.001$; **** - $p < 0.0001$.

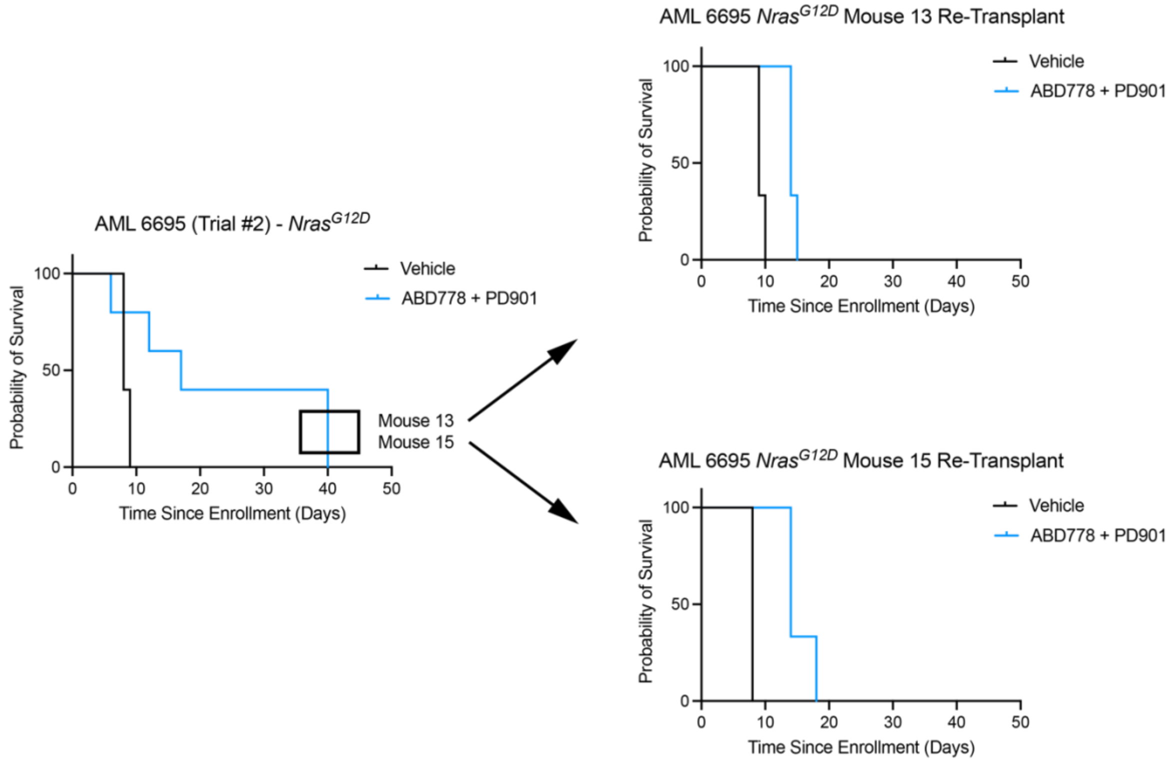

Extended Data Figure 8. Refractory AMLs that emerge after combination treatment exhibit intrinsic drug resistance. The left panel shows the survival of 5 recipient mice that were transplanted with *Nras*^{G12D} AML 6606 and treated ABD778 + PD901 (blue line; n =5). Leukemia cells collected at euthanasia from the two recipients shown in the black box were indpendently injected into secondary recipients and re-treated with either control vehicle or ABD778 + PD901 (n = 3 mice per group). The right panel shows the survival of these mice.

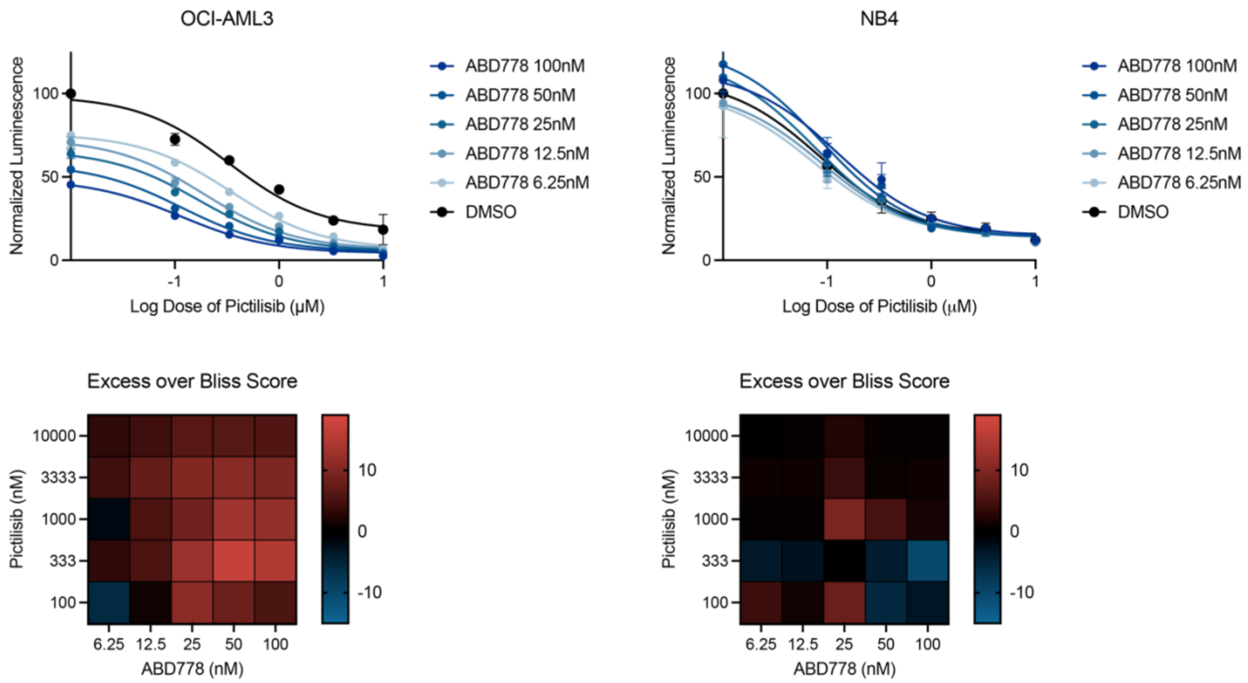

Extended Data Figure 9. Synergistic activity of ABD778 and pictilisib in AML cell lines expressing N-Ras^{G12D}. Bliss independence analysis demonstrating synergistic growth inhibition over a broad range of ABD778 and pictilisib concentrations in *NRAS*-mutant OCI-AML3 (left), but not *KRAS*-mutant NB4 (right), AML cells. Heatmaps display calculated synergy scores from strongly positive (red) to negative (blue). These data were generated in triplicate and were replicated in at least two additional independent experiments.

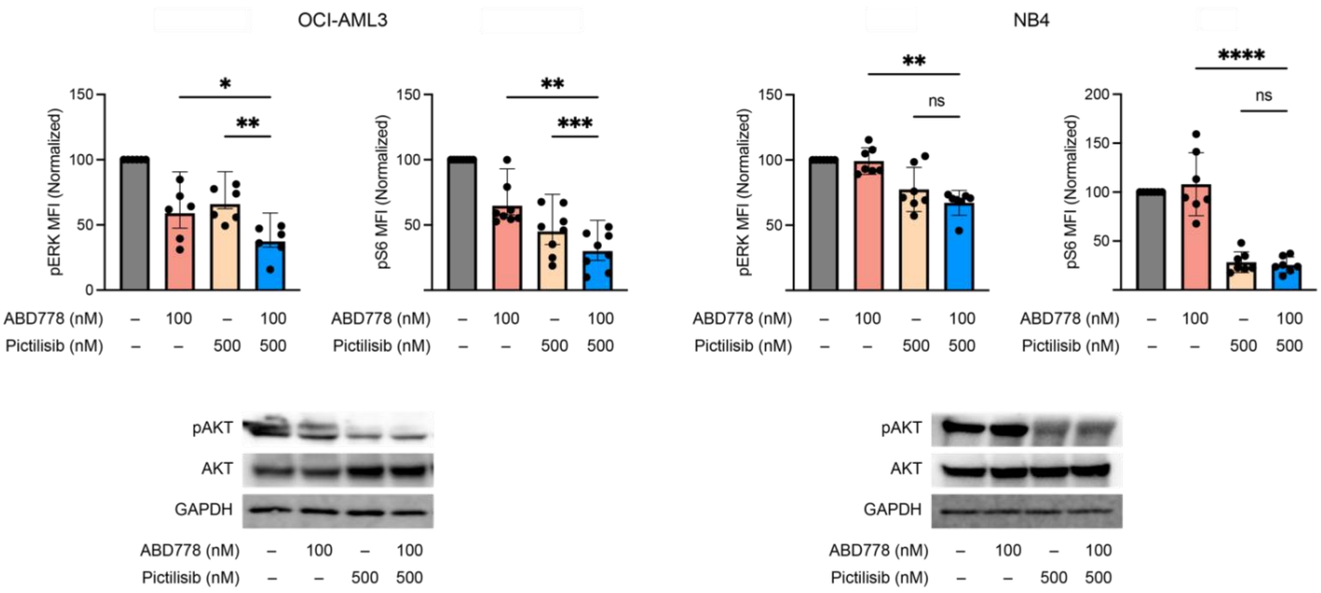

Extended Data Figure 10. Biochemical analysis of OCI-AML3 and NB4 AML cells that were exposed to DMSO vehicle, ABD770, pictilisib, or both drugs. Top**.** Phosphorylated ERK **(**pERK) and S6 (pS6) levels were measured using phospho-flow cytometry in OCI-AML3 (left) and NB4 (right) cells that were exposed to ABD778 and/or pictilisib for 4 hours at the doses shown. Mean fluorescence index (MFI) values were normalized to 100% of the DMSO control and pooled for statistical analysis (n = 6-8; **** - $p < 0.0001$). Bottom**.** pAKT levels were measured by Western blotting in OCI-AML3 (left) and NB4 (right) cells that were exposed to DMSO, ABD778, PD901, or both drugs. The data presented were generated in (phospho-flow) or are representative of (Western blots) at least three independent experiments.

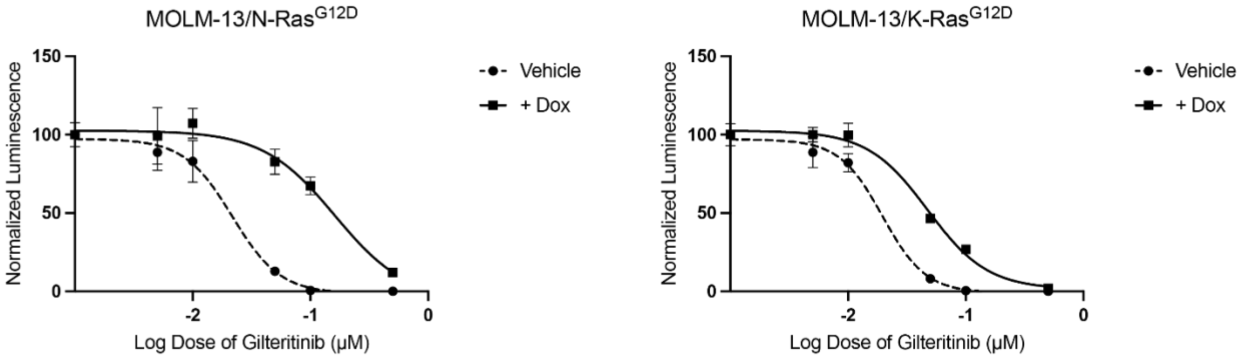

Extended Data Figure 11. Sensitivity of parental MOLM-13 cells and of cells engineered to express oncogenic N-Ras^{G12D} or K-Ras^{G12D} to giltiritinib. MOLM-13 cells were treated with doxycycline (Dox) for 24 hours to induce exogenous N-Ras^{G12D} (left) or K-Ras^{G12D} (right) expression and then exposed a range of gilteritinib doses for 72 hours. Proliferation was assessed by Cell Titer-Glo.

Extended Data Table 1

Mutation	Cytokine	Drug	HM4483	HM1989	HM2491	Average
NRAS	None	DMSO	6, 5	0, 0	16, 10	6.2
		ABD778	0, 0	0, 0	7, 6	2.2
	GM-CSF	DMSO	23, 16	7, 7	57, 36	24.3
		ABD778	2, 3	0, 0	29, 31	10.8

Mutation	Cytokine	Drug	HM4208	HM1959	HM4210	Average
KRAS	None	DMSO	48, 64	6, 6	12, 8	24.0
		ABD778	45, 52	5, 7	5, 8	20.3
	GM-CSF	DMSO	236, 228	24, 24	41, 28	96.8
		ABD778	253, 214	29, 23	39, 40	99.7

Legend**.** Number of GFU-GM colonies formed by JMML patient samples harboring somatic *NRAS* (HM4483, HM1989, HM2491) or *KRAS* (HM4208, MH1959, HM4210) mutations in methylcellulose medium, in the presence and absence of GM-CSF.

Extended Data Table 2

Leukemia	Driver Mutation	Drug and Dose	Vehicle Median Survival	PD901 Median Survival	Difference	P value
6695	$Nras^{G12D}$	PD901 5 mg/kg/day	10 days	21 days	11 days	0.0025
6606	$Nras^{G12D}$	PD901 5 mg/kg/day	16 days	26 days	10 days	0.01
21B	$Kras^{G12D}$	PD901 5 mg/kg/day	6 days	17 days	11 days	0.0027
6768	$Nras^{G12D}$	PD901 5 mg/kg/day	20 days	23 days	3 days	0.2
63A	$Kras^{G12D}$	PD901 5 mg/kg/day	8 days	19 days	11 days	0.0143

Legend. Responses of primary mouse AMLs to continuous, single agent treatment with PD901 (5 mg/kg/day) as reported in Burgess MR, et al.. *Blood*. 2014;124(26):3947-3955. and Burgess MR,, et al. *Cell*. 2017;168(5):817-829.e15. P values calculated by log rank (Mantel-Cox) test.

Extended Data Table 3

Leukemia	Driver Mutation	Drug and Dose	Vehicle Median Survival	PD901 Median Survival	Difference	P value
6695	*Nras*^{G12D}	PD901 2.5 mg/kg/day	11 days	19 days	8 days	0.0027
		ABD778 60 mg/kg/day	11 days	14 days	3 days	0.0027
		PD901 2.5 mg/kg/day + ABD778 60 mg/kg/day	11 days	32 days	21 days	0.0027
6768	*Nras*^{G12D}	PD901 2.5 mg/kg/day	18 days	34 days	16 days	0.0027
		ABD778 60 mg/kg/day	18 days	24 days	6 days	0.0027
		PD901 2.5 mg/kg/day + ABD778 60 mg/kg/day	18 days	38 days	20 days	0.0027
6606	*Nras*^{G12D}	PD901 2.5 mg/kg/day	11 days	19 days	8 days	0.0026
		ABD778 60 mg/kg/day	11 days	14 days	3 days	0.0026
		PD901 2.5 mg/kg/day + ABD778 60 mg/kg/day	11 days	41 days	30 days	0.0026
21B	*Kras*^{G12D}	PD901 2.5 mg/kg/day	4 days	14 days	10 days	0.0023
		ABD778 60 mg/kg/day	4 days	5 days	1 day	0.5485
		PD901 2.5 mg/kg/day + ABD778 60 mg/kg/day	4 days	10 days	6 days	0.0023
63A	*Kras*^{G12D}	PD901 2.5 mg/kg/day	9 days	22 days	13 days	0.0101
		ABD778 60 mg/kg/day	9 days	7 days	–2 days	0.2228
		PD901 2.5 mg/kg/day + ABD778 60 mg/kg/day	9 days	16 days	7 days	0.0514

Legend. Responses of cohorts of recipient mice (n = 5) that were transplanted with individual primary *Nras*^{G12D} or *Kras*^{G12D} AMLs and assigned to receive control vehicle, ABD778, PD901, or PD901 + ABD778. P values calculated by log rank (Mantel-Cox) test.

Extended Data Table 4

Leukemia	Driver Mutation	Mouse ID	Treatment	Gene and Mutation	Vehicle VAF	Drug Treated VAF
6606	*Nras*^{G12D}	13	PD901 + ABD778	*Nras*^{G12D}	0.61	0.90
				Asxl1^{I594fs}	0.48	0.48
				Kras^{A146T}	ND	0.06
		16	PD901	*Nras*^{G12D}	0.61	0.85
				Asxl1^{I594fs}	0.48	0.42
		18	PD901 + ABD778	*Nras*^{G12D}	0.61	0.94
				Asxl1^{I594fs}	0.48	0.48
				Map2k1$^{50\_63del}$	ND	0.07
				Braf^{G503E}	ND	0.18
		20	PD901 + ABD778	*Nras*^{G12D}	0.61	0.79
				Asxl1^{I594fs}	0.48	0.44
6768	*Nras*^{G12D}	13	PD901 + ABD778	*Nras*^{G12D}	0.40	0.55
				Cebbpa^{A83T}	0.45	0.50
		15	PD901 + ABD778	*Nras*^{G12D}	0.40	0.52
				Cebbpa^{A83T}	0.45	0.42
		18	PD901 + ABD778	*Nras*^{G12D}	0.40	0.49
				Cebbpa^{A83T}	0.45	0.48
				Braf^{E421V}	ND	0.03
6695	*Nras*^{G12D}	13	PD901 + ABD778	*Nras*^{G12D}	1.00	1.00
				Flt3D^{838E}	ND	0.03
				Fgfr1^{N506H}	ND	0.29
		15	PD901 + ABD778	*Nras*^{G12D}	1.00	0.99
				Pik3ca^{F977Y}	ND	0.04

Legend. Mutations were called from exome sequencing of genomic DNA that was isolated from bone marrow cells collected at euthanasia from mice treated with vehicle, PD901 or the PD901 + ABD778 combination. ND = not detected.

Extended Methods

Synthesis and Characterization of ABD778

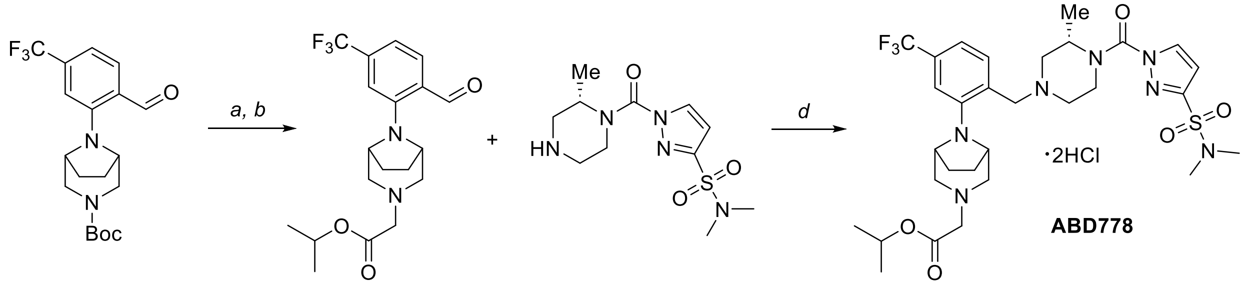

Step A: 2-((1R,5S)-3,8-diazabicyclo[3.2.1]octan-8-yl)-4-(trifluoromethyl)benzaldehyde hydrochloride

To a solution of *tert*-butyl 8-[2-formyl-5-(trifluoromethyl)phenyl]-3,8-diazabicyclo[3.2.1]octane-3-carboxylate (75.0 g, 195 mmol, 1.00 equiv) in DCM (200 mL) was added dropwise HCl in 1,4-dioxane (375 mL, 4 N) at 0 °C. The reaction was stirred at room temperature for 12 h before concentrating under reduced pressure to give 2-((1R,5S)-3,8-diazabicyclo[3.2.1]octan-8-yl)-4-(trifluoromethyl)benzaldehyde hydrochloride (64.0 g, crude) as a yellow solid. LCMS (ESI, *m/z*): 285 $[M+H]^+$.

Step B: isopropyl 2-{8-[2-formyl-5-(trifluoromethyl)phenyl]-3,8-diazabicyclo[3.2.1]octan-3-yl}acetate

A mixture of 2-((1R,5S)-3,8-diazabicyclo[3.2.1]octan-8-yl)-4-(trifluoromethyl)benzaldehyde hydrochloride (64.0 g, 225 mmol, 1.00 equiv) , isopropyl 2-bromoacetate (48.9 g, 270 mmol, 1.20 equiv) and K_2CO_3 (93.2 g, 675 mmol, 3.00 equiv) in DMF (1000 mL) was stirred at 100 °C for 12 h. The reaction was quenched with water (500 mL). The resulting solution was extracted with EtOAc (3 x 500 mL) and the combined organic layers were washed with brine (2 x 200 mL), dried over anhydrous Na_2SO_4 and concentrated under reduced pressure. The residue was chromatographed on a silica gel column with petroleum ether:EtOAc (8:1) to give isopropyl 2-{8-[2-formyl-5-(trifluoromethyl)phenyl]-3,8-diazabicyclo[3.2.1]octan-3-yl}acetate (65.8 g, 76% yield) as a yellow solid. LCMS (ESI, *m/z*): 385 $[M+H]^+$.

*Step C: isopropyl 2-[8-(2-{[(3S)-4-[3-(dimethylsulfamoyl)pyrazole-1-carbonyl]-3-methylpiperazin-1-yl]methyl}-5-(trifluoromethyl)phenyl)-3,8-diazabicyclo[3.2.1]octan-3-yl]acetate dihydrochloride (**ABD778**)*

To a stirred mixture of isopropyl 2-{8-[2-formyl-5-(trifluoromethyl)phenyl]-3,8-diazabicyclo[3.2.1]octan-3-yl}acetate (35.0 g, 91.0 mmol, 1.00 equiv) and (S)-N,N-dimethyl-1-(2-methylpiperazine-1-carbonyl)-1H-pyrazole-3-sulfonamide hydrochloride[1] (30.2 g, 100 mmol, 1.10 equiv) in MeOH (700 mL), was added AcOH (4 mL) at room temperature. The reaction was stirred for 12 h at room temperature prior to addition of $NaBH_3CN$ (17.2 g, 273 mmol, 3.00 equiv) at room temperature. The reaction was stirred at room temperature for 12 h before concentrating under reduced pressure. The residue was purified by reverse flash

chromatography with the following conditions: column, C18 column; mobile phase, MeCN in water, 60% to 70% gradient in 10 min; detector, UV 220 nm. The purifications were collected and lyophilized. The residue was dissolved into Et_2O (500 mL) and treated with HCl (17.9 mL, 2 N in Et_2O, 2 equiv). The resulting mixture was stirred for 2 h and concentrated to provide isopropyl 2-[8-(2-{[(3S)-4-[3-(dimethylsulfamoyl)pyrazole-1-carbonyl]-3-methylpiperazin-1-yl]methyl}-5-(trifluoromethyl)phenyl)-3,8-diazabicyclo[3.2.1]octan-3-yl]acetate dihydrochloride (ABD778) (27.3 g, 40.4% yield) as a white solid.

1H NMR (400 MHz, DMSO-d_6) δ 11.47 (s, 1H), 10.70 (s, 1H), 8.70 – 8.11 (m, 2H), 7.53 (d, J = 8.5 Hz, 1H), 7.23 (s, 1H), 6.91 (d, J = 2.7 Hz, 1H), 5.19 – 4.45 (m, 6H), 4.35 (s, 3H), 4.12 (s, 2H), 3.87 (s, 3H), 3.72 - 3.57 (m, 3H), 3.04 (s, 1H), 2.74 (s, 6H), 2.41 – 2.03 (m, 3H), 1.94 (d, J = 42.3 Hz, 1H), 1.57 (s, 3H), 1.25 (d, J = 6.2 Hz, 6H); 13C NMR (101, MHz, DMSO-d_6) δ 165.56, 150.42, 149.50, 135.14, 133.27, 130.96, 130.63, 128.29, 125.58, 122.87, 120.32, 120.16, 115.37, 108.70, 70.13, 60.64, 58.63, 57.85, 55.37, 54.61, 53.43, 52.06, 38.06, 26.04, 25.81, 21.90, 16.61; HRMS (m/z) [M+H]+ calcd. for $C_{30}H_{43}N_7O_5F_3S$, 670.2998; found 670.2995.

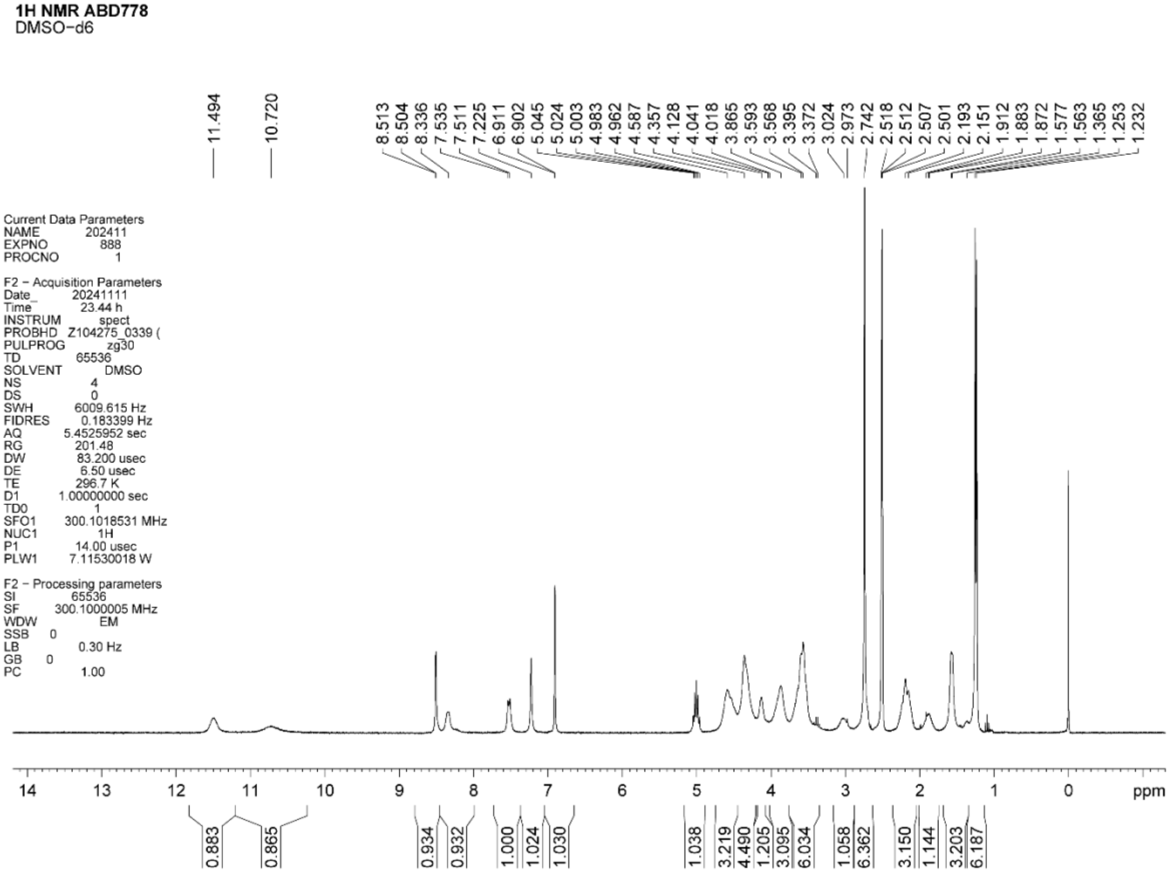

13C NMR ABD778
DMSO-d6

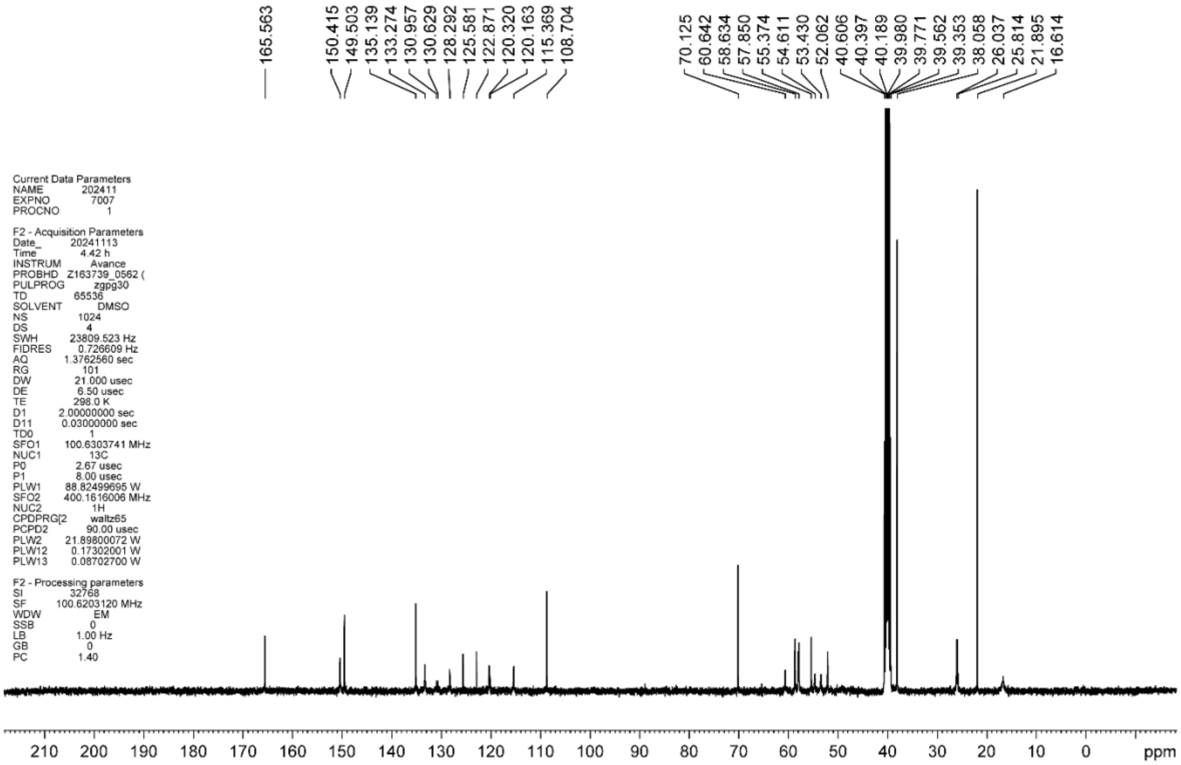

Supplement: Supplement 1 [file media-1.pdf]
